# Bionic natural small molecule co-assemblies towards targeted and synergistic Chemo/PDT/CDT

**DOI:** 10.1186/s40824-023-00380-z

**Published:** 2023-05-09

**Authors:** Shiyao Fu, Mingao Wang, Bin Li, Xu Li, Jianjun Cheng, Haitian Zhao, Hua Zhang, Aijun Dong, Weihong Lu, Xin Yang

**Affiliations:** 1 School of Medicine and Health, Harbin Institute of Technology, No. 92, West Dazhi Street, Nangang District, Harbin, 150001 China; 2 School of Chemistry and Chemical Engineering, Harbin Institute of Technology, No.92, West Dazhi Street, Nangang District, Harbin, 150001 China; 3 Department of Nephrology, the First Affiliated Hospital of Harbin Medical University, No. 23 Youzheng Street, Nangang District, Harbin, 150001 China; 4 Academician Workstation, Jiangxi University of Traditional Chinese Medicine, 1088 Meiling Street, Wanli District, Nanchang, 330004 No China; 5 Department of Ophthalmology, the Second Hospital of Jilin University, Nanguan District, No. 4026 Yatai Street, Changchun, 130041 China; 6 Chongqing Research Institute, Harbin Institute of Technology, Yubei District, No. 188 Jihuayuan South Road, Chongqing, 401135 China

**Keywords:** Triterpenoids, Co-assemblies, 4T1 cell membrane, Synergistic cancer treatment

## Abstract

**Background:**

Multi-component nano-delivery systems based on chemotherapy (chemo)- photodynamic therapy (PDT)- chemodynamic therapy (CDT) have gained increased attention as a promising strategy to improve clinical outcomes in cancer treatment. However, there remains a challenge in developing biodegradable, biocompatible, less toxic, yet highly efficient multicomponent nanobased drug delivery systems (DDS). Here, our study presents the screening and development of a novel DDS based on co-assemblies natural small molecule (NSMs). These molecules (oleanolic acid, and betulinic acid) are combined with photosensitizers Chlorine6 (Ce6) and Cu^2+^ that are encapsulated by tumor cell membranes. This nanocarrier encapsulated in tumor cell membranes achieved good tumor targeting and a significant improvement in tumor accumulation.

**Methods:**

A reprecipitation method was used to prepare the co-assembled nanocarrier, followed by the introduction of Cu^2 +^ into the DDS (OABACe6 NPs). Then, by wrapping the surface of NPs with the cell membranes of 4T1 which is a kind of mouse breast cancer cells (CM@OABACe6/Cu NPs). and analysis of its structure and size distribution with UV–Vis, XPS, FT-IR, SEM, TEM, and DLS. The synergistic effects of in vitro chemotherapy, CDT and PDT and targeting were also validated by cellular and animal studies.

**Results:**

It was shown that CM@OABACe6/Cu NPs achieved good tumor targeting and a significant improvement in tumor accumulation. In the composite nano-assembly, the NSMs work together with the Ce6 to provide effective and safe chemo and PDT. Moreover, the effect of reduced PDT due to the depletion of reactive oxygen species (ROS) by excess glutathione (GSH) in the tumor can be counteracted when Cu^2 +^ is introduced. More importantly, it also confers CDT through a Fenton-like catalytic reaction with H_2_O overexpressed at the tumor site.

**Conclusions:**

By constructing CM@OABACe6/Cu NPs with homologous targeting, we create a triple synergistic platform for cancer therapy using PDT, chemo, and CDT. We propose here a novel combinatorial strategy for designing more naturally co-assembled small molecules, especially for the development of multifunctional synergistic therapies that utilize NSMs.

**Supplementary Information:**

The online version contains supplementary material available at 10.1186/s40824-023-00380-z.

## Introduction

As is known to all, cancerous cells can spread to many different tissues and organs, which making treatment very difficult [1, 2]. The clinical efficacy of traditional cancer chemotherapy (chemo) has been severely compromised by a tendency to develop drug resistance and highly toxic side effects. Photodynamic therapy (PDT) is a promising therapeutic modality for cancer treatment, and can be combined with chemotherapy to improve overall survival rates [9, 10, 12]. By using visible light and photosensitizers, PDT kills tumor cells in the presence of oxygen. PDT has low toxicity, high responsiveness, low invasiveness, and good selectivity compared with conventional therapies [3–6]. However, photosensitizers (PSs) generally encounter limitations in their application, including poor water solubility, low bioavailability and low selection toward tumors. Therefore, effective PDT demands an excellent drug delivery system (DDS) to be designed to assist.

To remedy these gaps, nanocarrier arrived from natural compounds (e.g., polysaccharides [7], protein [8]) have become popularly used to construct drug delivery systems (DDSs) for the low toxicity, good biocompatibility, and long blood retention time [9]. Among them, the nanocarrier derived from natural small molecules (NSMs) with self-assembly function have emerged as a promising approach to treating cancer in recent years [10–13]. Some triterpenic acid [14], diterpenic acid [15] and sterol [16] of NSMs can be assembled to form nanocarriers possessed excellent bioactivity (e.g., anticancer [17, 18], antioxidant [19]) and biocompatibility. However, most NSMs cannot self-assemble into suitable nanocarrier morphologies to meet DDSs requirements. In order to control the physical dimensions and morphologies of NSMs nanocarrier, a supramolecular co-assembly strategy was utilized. Using the co-assembly strategy [20, 21], it is possible to create a multifunctional nano-delivery platform between different molecules of NSMs-based with synergistic antitumor effects. This strategy enables greatly expand the varieties and scopes of NSMs that was used to create multi-functional nanocarriers. However, due to the complexity of tumor pathogenesis, it often is difficult to achieve desirable therapeutic effect with single chemotherapeutic approach. Therefore, the use of multiple synergistic therapies instead of single therapy has become a fundamental part of cancer treatment in recent [22–28].

Chemodynamic therapy uses the tumour microenvironment to activate the Fenton reaction (Fenton-like reaction), which produces strongly oxidising hydroxyl radicals for tumour-specific treatment. Some metal ion-based nanomaterials show great potential because of their ability to generate ROS through fenton-like reactions, such as copper ion (Cu^2 +^). More importantly, the reaction rates are much greater than those of the Fenton reaction based on iron, under suitable circumstances. Meanwhile, Cu^2 +^ is the third most abundant transition metal ion in the human body, plays a vital role in the biological system. As an essential trace element, Cu^2 +^ has unique advantages in tumour therapy due to its active chemical properties and high catalytic capacity [29–31]. There is a general belief that the tumor microenvironment (TME) plays important role in tumor progression, invasive metastasis, and drug resistance, as well as the source of origin and residence of cancer cells [32, 33]. However, excessive GSH within the TME depletes ROS, which results in decreased efficacy of PDT. Stimuli-responsive anticancer therapies using nanocarriers containing metal ions (i.e., Cu^2 +^) are proving to enhance the efficacy of PDT by consuming excess intracellular GSH through redox reactions [34]. Overexpressed H_2_O_2_ in tumors undergo Fenton-like catalysis with Cu^2 +^ for CDT [35–37]. Therefore, Multiple synergistic therapies based on Chemo-PDT-CDT nano-delivery systems have emerged as a strategy for improving the clinical efficacy of cancer treatment.

It is one of the major challenges to design a nano-DDS with an active targeting capability in biomedicine, which provide effective active transport of drugs to specific locations as well as therapeutic properties. As tumor-targeting properties, nanocarriers encapsulated on cell membranes have better biodegradability, lower toxicity, lower immunogenicity, and higher biocompatibility than individual NPs [38, 39]. In addition, complex nanocarriers utilizing cell membranes as bionics can circulate for longer periods without being detected and eliminated by the immune system [40]. They can also be transported to specific sites with high efficiency and active transport, allowing for higher accumulation at the tumor site. 4T1 (mouse breast cancer cells) is a superficial cancer cell that maximises the therapeutic effect of PDT. We therefore chose 4T1 cell membranes to construct nanocarriers with homologous targeting [46].

**Scheme. 1:**
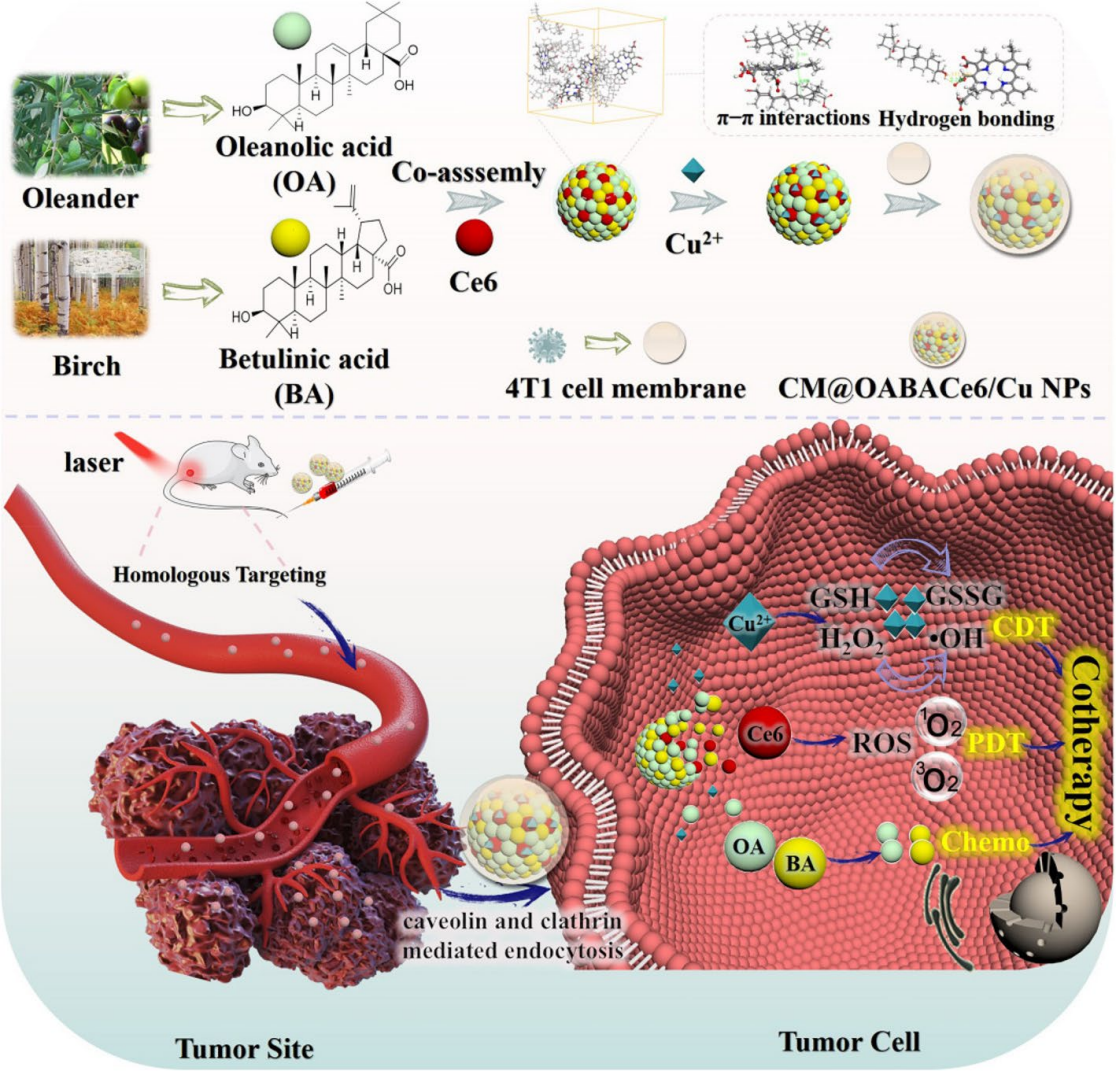
Illustration of the synthesis process for CM@OABACe6/Cu nano-assemblies that have PDT, CDT, and Chemo effects

This article implements a new synergistic strategy (Scheme 1) to improve the safety and efficacy of cancer treatment. The nanocarriers formed from co-assembly of two-component triterpenoids (oleanolic acid, and betulinic acid) were screened as chemotherapeutic agents and combined with photodynamic therapy (PDT). As NSMs are co-assembled with photosensitizer (Ce6), multifunctional DDSs are obtained that can exert synergistic effects when combined with different therapeutic approaches [41]. Then introducing Cu^2 +^ to the DDSs, the composite nano-assembly can both consume excess GSH at the tumor site and exert the ability of CDT. Finally, by wrapping the surface of NPs with the cell membranes of 4T1 which is a kind of mouse breast cancer cells, the NPs can target and accumulate at the tumor site due to the homing property of cancer cells [42], and further strengthen the effectiveness of tumor therapy derived from the combination of Chemo-CDT-PDT. A facile method is constructed for a synergistic oncology treatment platform with actively targeted natural nano-DDSs and provides a promising combination strategy that extends the medical applications of NSMs, in this study.

## Material and methods

### Material

Oleanolic acid (OA), Ursolic acid (UA), Betulinic acid (BA), Glycyrrhetinic acid (GA), and Betulic acid (Bet) were purchased from Nanjing Spring & Autumn Biological Engineering Co., Ltd (Nanjing, Jiangsu China). Chlorin Ce6 was purchased from Jinan Daen Pharmaceutical Technology Co., Ltd (Jinan, Shangdong, China). CuCl_2_ 2H_2_O was purchased from Aladdin Chemical Co. A membrane protein extraction kit was purchased from Sangon Biotech Co. GSH and a GSSG assay kit was purchased from Beyotime Co.

3-(4,5-Dimethylthiazol-2-yl)-2,5-dipheny-itetrazolium bromide (MTT) and a Singlet Oxygen Sensor Green (SOSG) were purchased from the Sigma-Aldrich Chemical Company. Methylene blue, 4,6-diamino-2-phenyl-indole (DAPI), and dichlorofluorescin diacetate (DCFH-DA) were purchased from Alfa Aesar Chemical Company. Calcitanin AM, Propidium iodide (PI), Annexin V-FITC/PI, glutaraldehyde, RIPA lysis buffer, chlorpromazine hydrochloride, chloroquine phosphate, nystatin, and amiloride (EIPA) were obtained from Beijing Solarbio Science & Technology Co., Ltd. Penicillin–streptomycin, fetal bovine serum (FBS), and RPMI 1640 medium were purchased from Sigma Gibco (Grand Island, NY, USA). Mouse breast cancer cell line (4T1) was obtained from the Shanghai Institute of Biochemistry (Shanghai, China).

### Preparation of Co-assemble triterpenoids NPs and the Triterpenoid-loaded Ce6 NPs

These nanocarriers were prepared using a reprecipitation method.The concentrations were selected on the basis of previous work in our laboratory [46]. In brief, the active natural product (OA, UA, BA, GA, or Bet) was dissolved in DMSO (33 mM) and subsequently mixed with 10 μL of OA and BA (v:v, 1:1). The solution was quickly added to 1 mL of distilled water. The OABA NPs were collected by centrifugation at 10,000 g for 20 min at 4℃. The NPs were washed twice with double-distilled water. Other combinations of nanocarriers were prepared similarly.

To prepare triterpenoid-loaded Ce6 NPs (OABACe6), 10 μL of Ce6, OA and BA in DMSO solution (33 mM) (v:v:v, 1:1:1) were mixed. The triterpenoid-loaded Ce6 NPs (UA, BA, GA, Bet, and Lup) were prepared in a similar way as described above.

### Morphological evaluation of NPs

The morphology of NPs was observed using a Quanta 200FEG scanning electron microscope (SEM) at 2 kV, 10 mA, spot size 3.5, and SE mode.

### Activity screening

The cytotoxicity of OAUA, OABA, OAGA, UABA, or UAGA NPs were determined using the MTT assay, and a mouse mammary cell line (4T1 cell) was selected. Briefly, cells (200 μL, 1 × 10^5^ cells/mL) were seeded in a 96-well plate and incubated for 24 h at 37℃. Subsequently, the cell culture medium was removed and a fresh medium containing various concentrations of OAUA, OABA, OAGA, UABA, or UAGA NPs (0–300 μg/mL) was added. The plate was incubated for 24 h. MTT (100 μL, 0.5 mg/mL) was added to the well and cultured with cells for 4 h. The medium was discarded and the MTT formazan crystals were dissolved with 200 μL DMSO. The absorbance of each well was measured using a microplate reader at 492 nm. Cell viability was calculated using the following formula.

1 $$Cell\;vialibilty\;\left(\%\right)\;=\frac{A_{sample}-A_{blank}}{A_{control}-A_{blank}}\;\mathrm x\;100\mathit\%$$

Relative parameters are defined as follows: A_sample_ is the cells treated with the drug. A_control_ is the cells treated with the medium. A_blank_ is the blank wells treated with PBS.

### Molecular Simulations of NPs Formation

The minimize and dynamics programs in the Discover Module of the Materials Studio 8.0™ software package were used for molecular dynamics (MD) simulation. The energy minimization of the three molecules, i.e., OA, BA, and Ce6 was carried out, and then MD simulations were done. The system for OA, BA, and Ce6 molecules in the water box was determined. The density of the modeling boxes was set to 1 g•cm^−3^. OA (8), BA (8), Ce6 (4), and 1000 water molecules were used in the simulation box. The COMPASS force file was used. Then the system temperature was set to 298 K in the MD run and the ensemble was set to NVT. The iteration step time was chosen as 1 fs, the trajectories were saved every 5 steps, and 5 ns of trajectories were obtained. A Berendsen thermostat was used, and the particle mesh was used to calculate electrostatic interactions.

### Preparation of a Cu^2+^-induced OABACe6 Assembly

Briefly, the OABACe6 NPs (0.41 mg) and CuCl_2_·2H_2_O (0.13 mg) were separately dissolved in 1 mL of distilled water. The CuCl_2_ was added to the NPs solutions. The mixtures were stirred at room temperature for 3 h with gentle stirring. Then, the NPs (OABACe6/Cu NPs) were collected by centrifugation at 10,000 g for 20 min at 4℃ and lyophilized for storage for a maximum of 8 wk.

### Preparation of CM@OABACe6/Cu NPs

Cell membrane (CM) material was obtained from cancer cells (i.e., 4T1 mouse breast cancer cell line) with the membrane protein extraction kit [42], and CM (typically from ~ 10^7^ cells) was used for 0.5 mg OABACe6/Cu NPs. The cell membranes were added to the NP solution using a Vortex for 1 min. Then, the mixture was subjected to probe ultrasound (100 W, 3 min, 10 s on, and 10 s off). The CM@OABACe6/Cu NPs were collected using centrifugation at 10,000 g for 20 min at 4℃.

### Particle Size Distribution and ζ-Potential

The particle size distribution and ζ-potential of these NPs were measured using dynamic light scattering (Malvern Instruments, Malvern, Worcestershire, UK).

### Morphological and elemental analysis

Morphology of the prepared NPs was characterized using a Tecnai G2 20 TWIN transmission electron microscope (TEM) and a Quanta 200FEG scanning electron transmission (SEM) at 20 kV. The elements of the prepared NPs were analyzed using X-ray photoelectron spectroscopy (XPS).

### Encapsulation Efficiency (EE)

The concentration of Ce6 in NPs was determined using high-performance liquid chromatography (HPLC). A reversed phase TC-C18 column (4.6 mm i.d. × 250 mm, Agilent Technologies, USA) was used as a column. The mobile phase was water (0.2% phosphoric acid) and acetonitrile (v:v, 40:60) at 1.0 mL/min, while the detection wavelength was 402 nm. The Ce6 encapsulation efficiency (EE) of various formulations was calculated using the following equation:

2 $$EE\mathit\;\mathit{\left(\%\right)}\mathit\;\mathit=\mathit\;\frac{\mathit{\left({W_{total\;Ce6}\;-W_{free\;Ce6}}\right)}}{{\mathit W}_{\mathit t\mathit o\mathit t\mathit a\mathit l\mathit\;\mathit C\mathit e\mathit6}}\mathit\;\mathrm x100\mathit\%$$

where W_total Ce6_ represents the total small molecules in the NP and W_free Ce6_ represents the amount of free small molecules in the filtrate.

### Stability

The stability of OABACe6 NPs and CM@OABACe6/Cu NPs in PBS and RPMI1640 medium was investigated by measuring the change in particle size on days 1, 2, 3, 4, 5, 6, and 7.

### Fluorescence quenching effects

OABACe6 and OABACe6/Cu NP (200 μg/mL) was dissolved in double distilled water. The fluorescence intensity (λ_EX_ = 405 nm, λ_EM_ = 650 nm) was determined using a fluorescence spectrophotometer. To investigate whether the introduction of Cu^2+^ leads to fluorescence quenching of Ce6.

### UV spectrum scanning

Ce6, OA-BA, OABACe6, OABACe6/Cu, and CM@OABACe6/Cu NPs were prepared with PBS (pH 7.4). UV − vis spectra were obtained using a TU-1900 PERSEE spectrometer (Beijing Purkinje General Instrument Co., Ltd, Beijing, China). The full scans (200 to 800 nm) were done for free Ce6, OA-BA, OABACe6, OABACe6/Cu, and CM@OABACe6/Cu NPs.

### Stimuli-responsive disassembly of CM@OABACe6/Cu NPs

CM@OABACe6/Cu NPs (2 mg/mL) were dissolved in various concentrations of GSH (2, 1.5, 1.25, 1, 0.5, 0.25, 0.1, 0.05, and 0 mM) and PBS (pH = 6.5) and incubated in a shaker (150 rpm) for 5 min at 37℃. The fluorescence intensity of Ce6 (λ_EX_ = 405 nm, λ_EM_ = 650 nm) was determined.

CM@OABACe6/Cu NPs (2 mg/mL) were dissolved in GSH (2 mM) and PBS (pH = 6.5) and incubate in the shaker for 0, 1, 2, 3, 4, 5, 10, 15, and 20 min at 37℃ to determine a time course. The fluorescence intensity of Ce6 was determined to examine whether disassembly occurred.

### Detection of GSH/GSSG Ratio

GSH (200 μM) was added to PBS (pH 6.5). OABACe6, OABACe6/Cu, and CM@OABACe6/Cu NP were dispersed in PBS (400 μg/mL) and incubated with shaking (150 rpm) for 3 h at 37℃. The GSH/GSSG ratio was measured using the GSH and GSSG assay kit following the manufacturer’s instructions. Determination was performed by a UV spectrophotometer.

### ^1^O_2_ Induced by CM@OABACe6/Cu NPs

The singlet oxygen sensor green (SOSG) has a high selectivity for singlet oxygen (^1^O_2_). CM@OABACe6/Cu NPs (20 μg/mL) and OABACe6 NPs (10 μg/mL) were dispersed in PBS (pH 6.5) with or without addition of GSH (200 μM). Solutions were incubated with shaking (150 rpm) for 3 h at 37℃, and SOSG (5 μM) was added. Then the sample was irradiated using a laser (660 nm, 0.6 W/cm^2^). The fluorescence intensity (λ_EM_ = 532 nm, λ_EX_ = 488 nm) was determined to examine the production of ^1^O_2_.

### Detection of OH using Coumarin (COU)

COU can be used as a fluorescent probe for OH. It is transformed into 7-OH COU after reaction with OH. NPs were dispersed in PBS (pH 6.5). Then COU and H_2_O_2_ were added to a final concentration of 2 mM and 1 mM, respectively (the final carrier concentration was 40 μg/mL). Other materials used were as follows: 1) COU + H_2_O_2_. 2) COU + CM@OABACe6/Cu NPs. 3) H_2_O_2_ + COU + CM@OABACe6/Cu NPs. 4) COU + OABACe6 NPs, and 5) H_2_O_2_ + COU + OABACe6 NPs. All samples were incubated with shaking for 3 h at 37℃ (150 rpm). The fluorescence intensity (λ_EX_ = 350 nm, λ_EM_ = 460 nm) was determined to examine the production of ^1^O_2_.

### Cell culture

Mouse breast cancer cell line (4T1) was obtained from the Shanghai Institute of Biochemistry (Shanghai, China). 4T1 was cultured in RMPI-1640 supplemented with 1% penicillin: streptomycin and 20% FBS at 37℃ with 5% CO_2_.

### Cell uptake

The cellular uptake of NPs was done using the 4T1 cell lines. Briefly, cells (1 × 10^6^ cells per mL) were seeded in a 6-well plate and incubated for 24 h at 37℃. The cell culture medium was removed and a fresh medium containing free Ce6, OABACe6 NPs, and CM@OABACe6/Cu NPs (the concentration of Ce6 was 2 μg/mL) was added and incubated for 0, 5, and 30 min for 3 h. The uptake of different NPs by 4T1 cells was observed at different time points with inverted fluorescence microscopy.

### Investigating the mechanism of cell  internalization

The pathway for the internalization of CM@OABACe6/Cu NPs into cells was studied. The low-temperature condition was determined as 4℃, and the endocytic inhibitors were chlorpromazine hydrochloride, chloroquine phosphate, nystatin, and EIPA. Briefly, cells 10^6^ cells per mL) were seeded in 6-well plates and incubated for 24 h at 37℃. Samples were divided into six groups: 1) cultured at 4℃, control, and all others 2) at 37℃, 3) with chlorpromazine hydrochloride, 4) chloroquine phosphate, 5) nystatin, and 6) EIPA. The cell culture medium was removed, washed three times with cold PBS, and a fresh medium containing appropriate concentrations of CM@OABACe6/Cu NPs (the concentration of Ce6 was 2 μg/mL) was added to the wells and incubated for 24 h. The samples were fixed using 4% paraformaldehyde for 15 min and the uptake of NPs by 4T1 cells was observed using an inverted fluorescence microscope.

### In vitro ROS detection

DCFH-DA is a fluorescent probe for ROS. Intracellular ROS could oxidize non-fluorescent DCFH to produce fluorescent DCF. Briefly, DCFH-DA was dispersed in RPMI-1640 medium (10 μM). Cells (1 × 10^6^ cells per mL) were seeded in 6-well plates and incubated for 24 h at 37℃. Cells were incubated with DCFH-DA for 30 min. The cells were washed six times with cold PBS. Samples were divided into six groups: 1) Incubated with RPMI-1640 medium for 4 h. 2) Incubated with RPMI-1640 medium for 4 h then 675 ± 10 nm laser (150 mW/cm^2^) irradiated for 10 min. 3) Incubated with CM@OABACe6/Cu NPs (the concentration of Ce6 was 2 μg /mL) for 4 h. 4) Incubated with CM@OABACe6/Cu NPs (the concentration of Ce6 was 2 μg /mL) for 4 h and washed three times with PBS, and 675 ± 10 nm laser (150 mW/cm^2^) irradiated for 10 min. 5) Incubated with free Ce6 (2 μg /mL) for 4 h. and 6) Incubated with free Ce6 (2 μg /mL) for 4 h and washed three times with PBS, and 675 ± 10 nm laser (150 mW/cm^2^) irradiated for 10 min. The samples were fixed with 4% paraformaldehyde for 15 min, and ROS production in 4T1 cells was observed with an inverted fluorescence microscope.

### MTT Assay

The cytotoxicity and PDT effects of OABACe6 NPs, OABACe6/Cu NPs, and CM@OABACe6/Cu NPs were determined using the MTT assay, and the mouse breast cancer cell line (4T1). Briefly, cells (200 μL, 1 × 10^5^ cells per mL) were seeded in a 96 well plate and incubated for 24 h at 37℃. Then, the cell culture medium was removed and a fresh medium containing various concentrations of OABACe6 NPs, OABACe6/Cu NPs, and CM@OABACe6/Cu NPs (the concentration of Ce6 was 0.1–10 μg/mL) was added. Samples were divided into two groups: laser and no-laser groups. The no-laser group was incubated for 24 h. The laser group was incubated for 4 h, laser-irradiated for 10 min, and incubated for 20 h. MTT solution (100 μL of 0.5 mg/mL) in PBS was added to the well and cultured with cells for 4 h. The medium was discarded and the MTT formazan crystals were dissolved with 200 μL DMSO. The absorbance of each well was measured using the microplate reader at 492 nm. Cell viability was calculated using the following formula:

3 $$Cell\;viability\;\left(\%\right)\;=\frac{A_{sample}-A_{blank}}{A_{control}-A_{blank}}\;\mathrm x\;100\;\%$$

Relative parameters are defined as follows: For A_sample_ the cells were treated with NP. A_control_ was the cells treated with the medium. A_blank_ was the blank wells treated with PBS.

### Live/Dead cell staining

Calcein-AM is a fluorescent staining reagent for labeling active cells, which can penetrate cells. Propidium iodide (PI) cannot cross the cell membrane of a living cell but can cross the dead cell membranes to reach the nucleus. A Calcein-AM/PI double stain kit was used to study the intracellular CDT efficacy of CM@OABACe6/Cu NPs. Briefly, cells (1 × 10^6^ cells per mL) were seeded in a 6 well and incubated for 24 h at 37℃. Samples were divided into four groups: 1) RPMI-1640 medium. 2) H_2_O_2_. 3) CM@OABACe6/Cu NPs. And 4) CM@OABACe6/Cu NPs (the concentration of Ce6 was 2 μg/mL) + H_2_O_2_. All samples were incubated for 24 h at 37℃. Then cells were collected by centrifugation as previously described and suspended in a solution containing Calcein-AM and PI for 15 min. Samples were fixed using 4% paraformaldehyde and observed using the inverted fluorescence microscope.

### Establishment of a tumor model

Female Balb-c (6–8 wk old, 18–22 g) were obtained from the Second Affiliated Hospital of Harbin Medical University (Harbin, Heilongjiang, China), and all animal experiments were carried out according to the protocol approved by the Institutional Animal Care and Use Committee (IACUC) of Harbin Medical University. 4T1 mouse mammary carcinoma cells (2.5 × 10^5^ cells per mL) were injected into the right flank of each mouse. Vernier calipers were used to determine the length and width of the tumors in mice. PDT was determined when the 4T1 tumor carrying mice reached about 50–100 mm^3^ in volume. The tumor volume (V) was calculated according to the formula:

4 $$V\mathit\;\mathit=\frac{L\mathit\;\mathrm x\mathit\;W^{\mathit2}}{\mathit2}$$

where L and W represent tumor length and width, respectively.

### Hemolysis analysis

Blood compatibility, the potential toxicity, and biosafety of CM@OABACe6/Cu NPs were evaluated using a hemolysis test. Briefly, mice blood was washed with cold PBS three times with centrifugation (1000 g, 10 min, 4℃), blood was diluted 10 × with PBS and divided into seven groups including controls, OABACe6/Cu NPs, and CM@OABACe6/Cu NPs at different concentrations (100, 200, and 400 μg/mL). Samples were added into tubes and incubated at 37℃ for 6 h, and the results of the hemolysis study were obtained using a smartphone camera. Observe the presence of erythrocytes deposited at the bottom.

### In vivo fluorescence imaging

For in vivo fluorescence imaging, CM@OABACe6/Cu NPs were injected into the tumor-bearing mice body through the tail vein. The mice were randomized into four groups (n = 3): 1) saline, 2) free Ce6 groups (Ce6 equivalent for all groups: 4.5 mg/kg), 3) CM@OABACe6/Cu NPs groups, and 4) CM@OABACe6/Cu NPs groups. The tail vein injection volume was 150 μL. Mice were killed by neck cutting method at 0, 2, 4, 8, 12, and 24 h for harvesting of tumors and main organs. The fluorescence signals of tumors and organs were obtained using the Maestro in vivo optical imaging system (Cambridge Research & Instrumentation, Inc.

### Screening of dose of administration

The effect of CM@OABACe6/Cu NPs dose on the anticancer effect, was studied using seven groups (*n* = 5) of mice: 1) saline group, 2) OABACe6/Cu NPs group (Ce6 equivalent: 2.5 mg/kg), 3) OABACe6/Cu NPs groups (Ce6 equivalent: 3.5 mg/kg), 4) OABACe6/Cu NPs groups (Ce6 equivalent: 4.5 mg/kg), 5) CM@OABACe6/Cu NPs groups (Ce6 equivalent: 2.5 mg/kg), 6) CM@OABACe6/Cu NPs groups (Ce6 equivalent: 3.5 mg/kg), and 7) CM@OABACe6/Cu NPs groups (Ce6 equivalent: 4.5 mg/kg). The NPs were injected into the body through the tail vein and were given once every other day. The tail vein injection volume was 150 μL. The mice in the light group were irradiated using the 675 ± 10 nm laser (150 mW/cm^2^) for 15 min after 6 h post-injection. The tumor volume and body weight were measured daily for 12 days.

### In vivo antitumor activity

To study the anticancer efficiency of CM@OABACe6/Cu NPs, the tumor-bearing mice were randomized into eight groups (*n* = 5): 1) blank group: PBS, 2) control group: Ce6 + Laser, 3) chemo group: OABACe6 NPs, 4) chemo + PDT group: OABACe6 NPs + Lase, 5) chemo + CDT group: OABACe6/Cu NPs, 6) chemo + CDT + PDT group: OABACe6/Cu NPs + laser, 7) targeted + chemo + CDT group: CM@OABACe6/Cu NPs, and 8) targeted + chemo + CDT + PDT group: CM@OABACe6/Cu NPs + laser (Ce6 equivalent: 4.5 mg/kg for all samples). The NPs are injected into the body through the tail vein, every other day, for a total of three injections. The laser treatment was the same as that in the previous section. The tumor volume and body weight were measured daily for 14 days after the initial treatment to assess the antitumor activity and toxicity of the drug carrier.

### Safety evaluation

To study the toxicity of CM@OABACe6/Cu NPs, the tissues and organs of tumor-bearing mice were stained at the end of the PDT treatment. The main organs including tumor, heart, liver, spleen, kidney, and lung were collected and stained with hematoxylin and eosin (H&E) and observed using an optical microscope. Comparisons were made with the blank group for each tissue and organ.

### Statistical analysis

All the data given in this study are represented as the mean value with standard deviation (mean ± SD) results from mumbles of independent experiments. The two-side student’s tests (SPSS 19.0 software, Chicago, IL, USA) were used to compare the statistical significance of the results. The value of *P* < 0.05 was considered as statistically significant (*p < 0.05 represents a significant difference and **p < 0.01 a very significant difference).

## Results and discussion

### Screening of bioactive platforms, preparation, and characterization of NP

NSMs have the unique self-assembly property that allows them to be assembled to construct drug carriers. Over the years, a large number of studies have shown that pentacyclic triterpenes have a wide range of pharmacological effects and biological activities, especially in anti-inflammatory, hepatoprotective, anti-tumor, and immunomodulatory aspects have shown intriguing pharmacological properties. Oleanolic acid (OA), Ursolic acid (UA), Betulinic acid (BA), Glycyrrhetinic acid (GA), and Betulic acid (Bet) are representative pentacyclic triterpenoids in various plants and fruits. OA and UA have hepatoprotective properties. BA, GA, and Bet have anti-tumour properties. Previous work in our laboratory results that NSMs can co-assemble with Ce6, this paper developed a three-component small molecule co-assembled nano-drug delivery system to provide multiple anti-tumor effects.

**Fig. 1:**
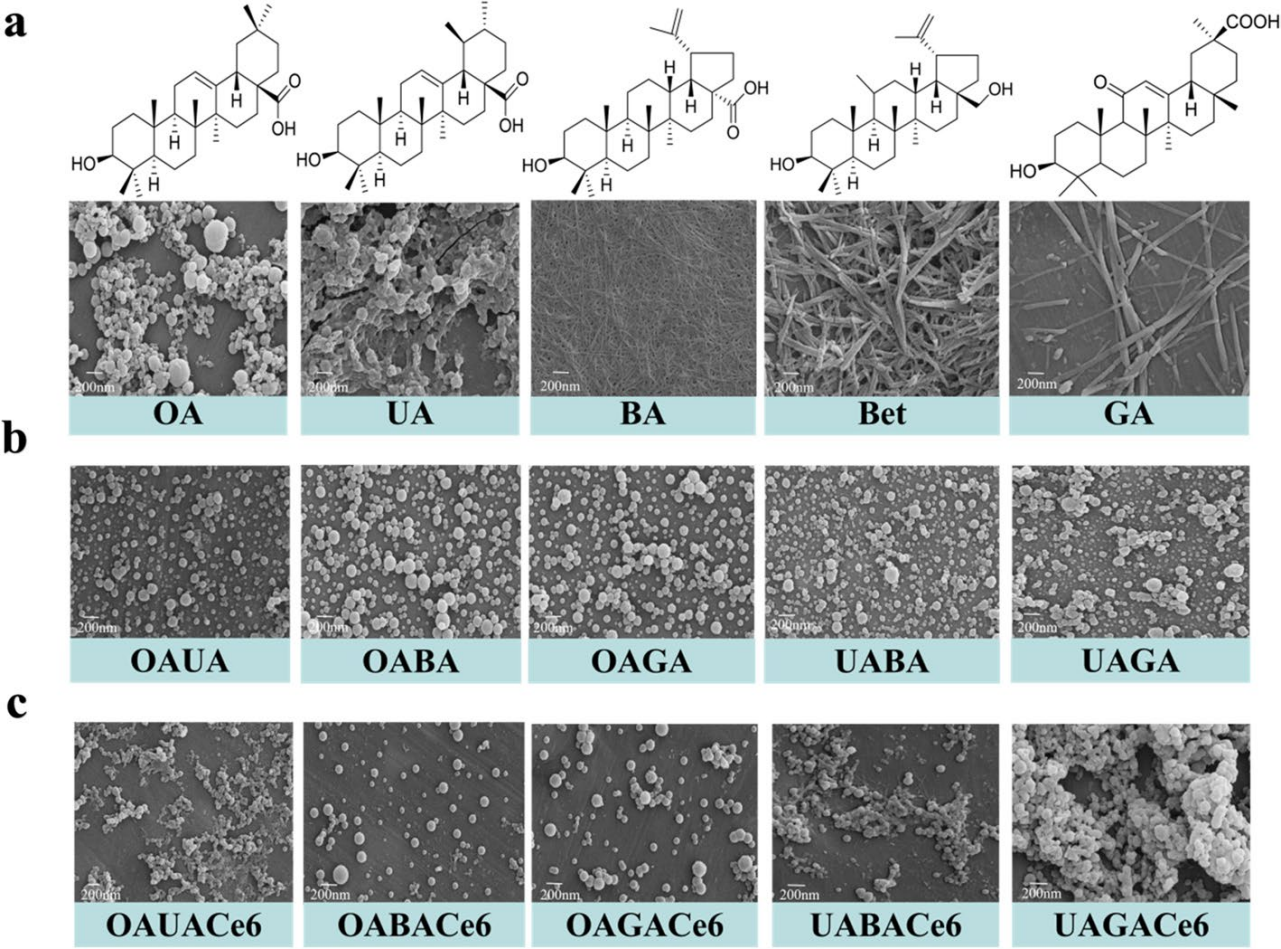
**a** Structure and corresponding SEM images of natural small molecules. Top: natural small molecule compound structure, bottom: SEM image of corresponding assembly structure. **b** SEM image of co-assembled NPs. **c** SEM image of co-assembled NPs loaded with Ce6

As mentioned above, we prepared nanocarriers by the reprecipitation method. Scanning electron microscopy (SEM) results showed that OA, and UA could form nanospheres, BA, GA, and Bet could form nanofibers, respectively (Fig. 1a). However, BA, GA and Bet form a self-assembled morphology that is not suitable for drug delivery. Previous studies have shown that two natural small molecules can co-assemble to form NPs with each other. Therefore, OA and UA were co-assembled with these three NSMs to improve the assembled morphology, and about ten formulations were obtained. In the SEM image results (Fig. 1b), several key combinations have attracted our attention, such as OAUA NPs, OABA NPs, OAGA NPs, UABA NPs, and UAGANPs. They combined to form small sized nanocarriers, while the other groups formed larger sized nanospheres or showed a mixture of the two structures, indicating that they did not combine together to form NPs or had large particle sizes (Fig. S1).

Subsequently, to investigate whether the structure of co-assembled nanocarriers was changed after the loaded of Ce6, the same method was employed to prepare co-assembled nanocarrier loaded with Ce6. The volume ratio chosen after screening was 1:1:1 (Table. S1). SEM showed that two combinations still formed nanospheres, such as OABACe6 NPs, and OAGACe6 NPs. However, the OAUACe6 NPs and UABACe6 NPs show fiber when co-assembled, while the UAGACe6 NPs show irregular clusters when co-assembled (Fig. 1c). This suggests that the addition of Ce6 does alter the morphology of the co-assembled NPs. There are some changes in the shape of several other groups (Fig. S1). After that, activity screening was performed on the five groups of co-assembled NPs (Fig. S2), among which OABA NPs showed the strongest activity. The optimal drug loading of Ce6 on OABA NPs was 3.28% (Table. S2-4). Therefore, based on these results, OABA NPs were selected as the preferred nanocarriers.

### Assembled mechanism of CM@OABACe6/Cu NPs

**Fig. 2:**
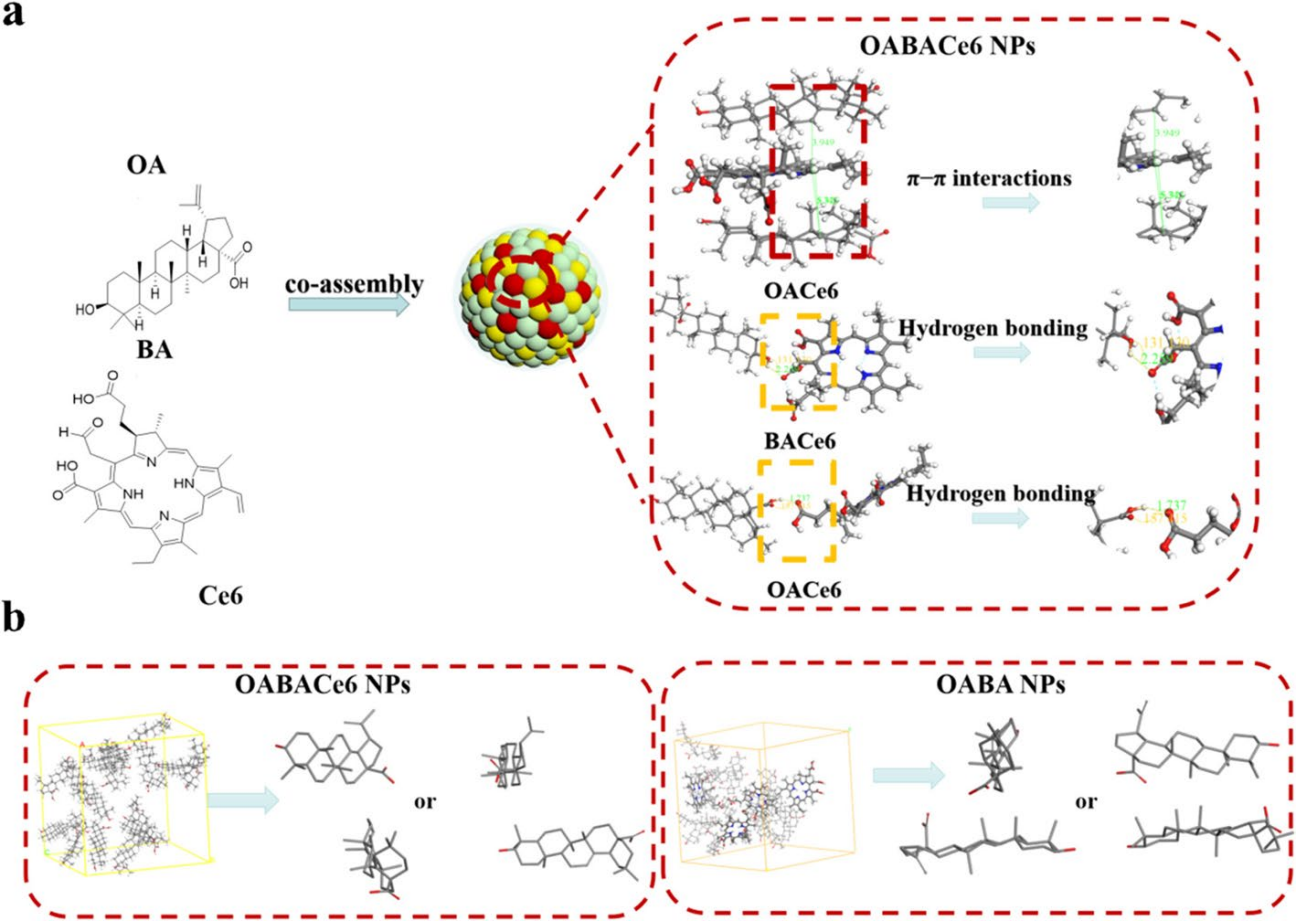
**a** Schematic representation of the possible co-assembly behavior of OABACe6 NPs and **b**) molecular stacking models of co-assembled OABA NPs and OABACe6 NPs

To further study the interaction mechanism between the carrier and the drug, we performed Molecular Dynamics (MD) simulation on OABACe6 NPs. The hydrophilic and hydrophobic surfaces showed that both OA and BA molecules matched with Ce6 molecules on hydrophilic surfaces (hydrogen bond donor and acceptant), the formation of hydrogen bond forces (Fig. 2a). OA and BA molecules tend to match the hydrophilic properties of Ce6, which increases the connectivity between the hydrogen bond donor and the hydrogen bond acceptor in the two molecules, and finally forms the hydrogen bond force (the bond length between OA and Ce6 is about 1.737 Å and the bond angle is about 157°; the bond length between BA and Ce6 is about 2.259 Å and the bond angle is about 131°). Meanwhile, the pyridine ring in Ce6 forms a π-π interaction with the double bond in OA (the bond length is about 3.949 Å). Then, the spatial conformation of the skeleton of OA and BA in each group was analyzed to explore the accumulation pattern between OA and BA molecules. In OABA NPs and OABACe6 NPs models, the spatial positions of the molecular skeletons of OA and BA were both vertical or trapezoidal configurations, which was conducive to the formation of hydrophobic interaction between the molecular skeletons (Fig. 2b) (Fig. S3). These results show that the two molecules capable of forming co-assembled NPs exhibit the same pattern of molecular binding and molecular stacking, i.e. vertical stacking spatial configurations capable of forming hydrophobic interactions.

### Characterization and analysis of CM@OABACe6/Cu NPs and Stimuli-Responsive Properties

**Fig. 3:**
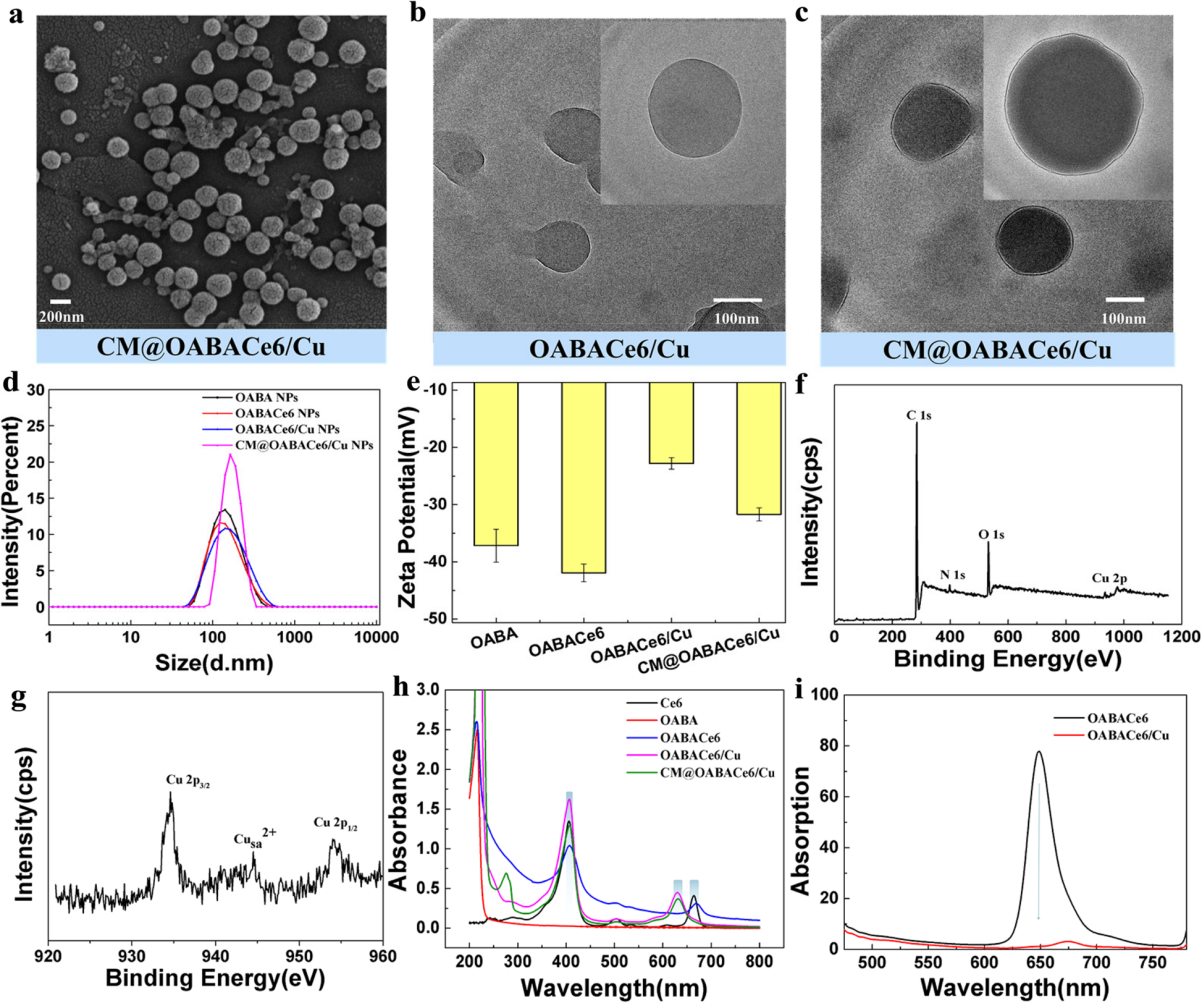
**a** SEM image of CM@OABACe6/Cu NPs, **b** TEM image of OABACe6/Cu NPs and **c**) TEM image of CM@OABACe6/Cu NPs. **d** Hydrodynamic diameter and **e**) ζ-Potential of OABA NPs, OABACe6 NPs, OABACe6Cu NPs, and CM@OABACe6/Cu NPs. **f** XPS survey spectrum and, **g**) high-resolution Cu 2p spectrum of CM@OABACe6/Cu NPs. **h** UV/Vis absorption spectra of Ce6, OABA NPs, OABACe6 NPs, OABACe6/Cu NPs, and CM@OABACe6/Cu NPs. **i** FL emission spectra of OABACe6 NPs and CM@OABACe6/Cu NPs

Cu^2+^ was introduced to endow OABACe6/Cu NPs with the ability of CDT and play the role of GSH consumption in the tumor. Through the central nitrogen atom of Ce6 it is possible to form complexes with metal ions. Thus, Cu^2 +^ can be introduced into NPs. Then, we provide an external force by physical means (ultrasound) to coat the cell membrane directly onto the surface of the NPs, which had a highly effective homologous targeting effect on tumors in vivo [45]. Subsequently, the surface state and molecular composition of the nanocarriers were characterized using SEM, X-ray photoelectron spectroscopy (XPS), size, ζ-potential, TEM, and UV/Vis absorption spectroscopy. The spherical shape and uniform size of CM@OABACe6/Cu NPs were observed by SEM, indicating that the NPs had been successfully assembled (Fig. 3a) (Fig. S4). TEM showed that CM@OABACe6/Cu NPs showed a double-layer membrane structure, showing the successfully coated 4T1 cell membrane on the surface of CM@OABACe6/Cu NPs (Fig. 3b, c). The ζ-Potential of OABA NPs and OABACe6 NPs decreased from -37.1 mV to -41.9 mV (Fig. 3d), which might be due to the introduction of -COOH of Ce6. Compared with OABACe6/Cu NPs, the ζ-Potential of OABACe6/Cu NPs increased from -37.1 mV to -22.8 mV, attributing to the positive charge effect of Cu^2+^. The surface charge of CM@OABACe6/Cu NPs was -31.7 mV, which is the negative charge on the surface of the cell membrane leading to the zeta potential reduction. The particle sizes of OABA NPs, OABACe6 NPs, and OABCe6/Cu NPs were 138.2 ± 2.3 nm, 147 ± 4.6 nm, and 151.4 ± 2.5 nm, respectively (Fig. 3e). The particle size of CM@OABACe6/Cu NPs increased to 174.5 ± 5.3 nm, which is due to the thickness of the 4T1 cell membrane, making its particle size increase significantly after wrapping the cell membrane. In order to see whether proteins of the 4T1 cell membrane were preserved intact after a series of treatments, we set up the 4T1 cell membrane group and CM@OABACe6/Cu NPs group. From the results, it can be seen that most of the membrane proteins of the CM@OABACe6/Cu NPs group were not lost during the sample preparation and the main protein bands were generally consistent with those of the 4T1 cell membrane (Fig. S5). XPS results confirmed the chemical composition of CM@OABACe6/Cu NPs (Fig. 3f). The spectra show that the characteristic peaks of C 1 s, N 1 s, O 1 s, and Cu 2P are 284.9 eV, 399.3 eV, 531.9 eV, and 934.2 eV, respectively. The High-resolution XPS spectrum of Cu 2p (Fig. 3g) reveals two major peaks at 934.6 and 954.2 eV, which are Cu 2p 3/2 and Cu 2p 1/2, respectively. In addition, a satellite peak at 938–948 eV was observed, which is typical for Cu^2+^ [43]. The presence of Cu^2+^ in CM@OABACe6/Cu NPs was verified. According to UV/Vis spectra, Ce6 has absorption peaks at 405 nm and 660 nm (Fig. 3h). After being assembled into OABACe6 NPs, the absorption peak of Ce6 shows a red-shifted UV absorption peak around 635 nm, which was due to the π-π conjugation effect between OA, BA molecular, and Ce6. After the introduction of Cu^2+^, the absorption peak of Ce6 show a blue-shifted. Nevertheless, after OABACe6 NPs complexed Cu^2+^, which weakened the conjugation of Ce6 because Cu^2+^ was an electron-withdrawing group. The UV/Vis results demonstrate the interaction forces between OA, BA, and Ce6 molecules, resulting in co-assembly. The fluorescence spectra showed that after assembly into CM@OABACe6/Cu NPs, FL at the best emission channel of Ce6 (Lex = 405 nm) was significantly quenched (Fig. 3i), which was primarily due to Cu^2 +^ induced OABACe6 NPs aggregation and electron/energy transfer process. These results confirmed that π − π stacking and hydrogen bonding may be the main driving forces for the formation of nanostructured OABACe6 NPs.

**Fig. 4:**
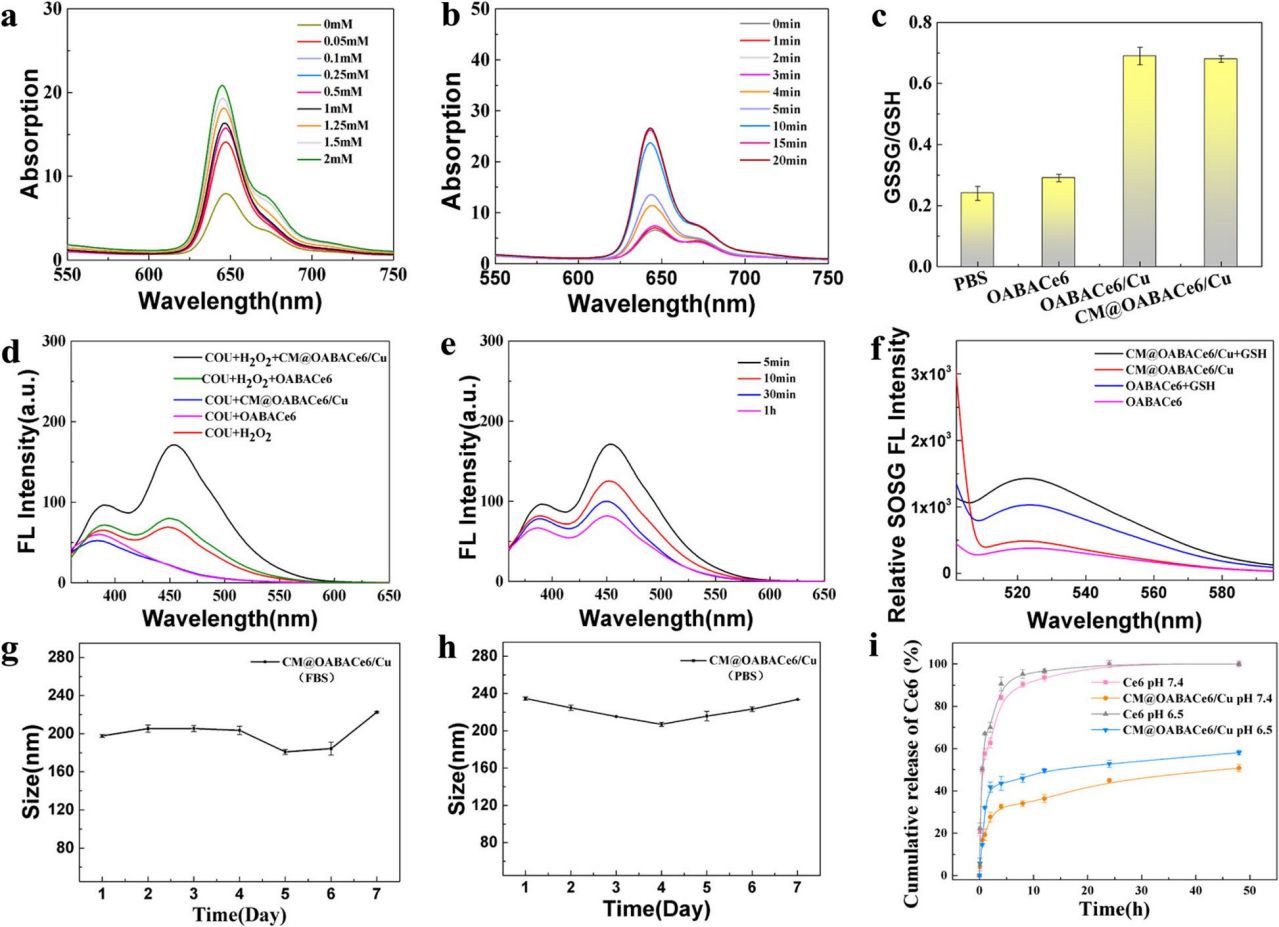
**a** FL intensity of CM@OABACe6/Cu NPs with GSH concentration (GSH concentration 0-2 mm). **b** FL intensity of CM@OABACe6/Cu NPs respectively (time 0–20 min) **c** The consumption rate of GSH after cultured with PBS, OABACe6 NPs, OABACe6/Cu NPS, and CM@OABACe6/Cu NPs, respectively **d**) The FL spectra of COU with H_2_O_2_, OABACe6/Cu NPs and CM@OABACe6/Cu NPs, and the FL spectra of COU with OABACe6/Cu NPs, and CM@OABACe6/Cu NPs, respectively. **e** FL emission spectra of SOSG in OABACe6 NPs and CM@OABACe6/Cu NPs after incubation with or without GSH and then exposure to laser irradiation. **f** FL emission spectra of SOSG in CM@OABACe6/Cu NPs after incubation with GSH and then exposure to laser irradiation, respectively (time 0.5 min-1 h). **g** CM@OABACe6/Cu NPs were stable at RPMI 1640 (with 20% FBS). **h** CM@OABACe6/Cu NPs were stable in PBS (pH 7.4). **i** In vitro release of CM@OABACe6/Cu NPs in pH 6.5 and pH 7.4 release media

The TME includes low pH, overexpressing GSH, and H_2_O_2_. Therefore, we investigated the response characteristics of CM@OABACe6/Cu NPs to TME stimuli. As shown in Fig. 4a, in the presence of PBS (pH = 6.5) and GSH (200 μg/mL), the fluorescence of Ce6 emitted from CM@OABACe6/Cu NPs gradually recovered with the increase of GSH concentration. However, the fluorescence of Ce6 gradually increased with time and remained constant after 10 min (Fig. 4b). Meanwhile, the electrostatic repulsion between metal ions and H + in an acidic medium will be enhanced, which will weaken the coordination ability between Cu^2+^ and Ce6, thus leading to the disassembly of CM@OABACe6/Cu NPs. In addition, it is known that GSH is highly expressed at tumor sites and ROS are scavenged due to excess GSH in vivo, thus decreasing the effectiveness of cancer treatment. Cu^2+^ can cause excess intracellular GSH to be depleted through redox reactions, which can be demonstrated by assaying the GSSG/GSH ratio in GSH solution with the GSSG/GSH kit. The results were shown in Fig. 4c. Compared with OABACe6/Cu NPs and PBS, the GSSG/GSH ratio observed in GSH solution after incubation with OABACe6/Cu NPs and CM@OABACe6/Cu NPs increased by about 3-folds. The results showed that the introduction of Cu^2+^ significantly increased the consumption capacity of GSH. Furthermore, Cu^2+^ was reported to be effective in catalyzing H_2_O_2_ and generating hydroxyl radicals (·OH) via Harber-Weiss and Fenton-like reactions even in weakly acidic media. Coumarin (COU) can be used as a specific probe for the ·OH production properties of CM@OABACe6/Cu NPs (i.e. COU can be used as a fluorescent probe of hydroxyl radicals and react with hydroxyl radicals to generate 7-COU, which has a characteristic FL peak near 460 nm). As depicted in Fig. 4d, the fluorescence intensity of the mixture of COU, H_2_O_2_ and CM@OABACe6/Cu NPs was stronger than that of the other controls, which verified the efficient response of H_2_O_2_ to generate ·OH and the CDT ability of CM@OABACe6/Cu NPs. Then the FL emission spectra of COU, H_2_O_2,_ and CM@OABACe6/Cu NPs mixtures with time were investigated. The FL emission intensity decreases with time. It was demonstrated that ·OH quenched with time (Fig. 4e). Then, the photosensitization of CM@OABACe6/Cu NPs was evaluated by using Singlet Oxygen Sensor Green (SOSG) as a fluorescent probe (Fig. 4f). The results showed that the FL emission of SOSG in the CM@OABACe6/Cu NPs dispersion with GSH was stronger than that without GSH at 660 nm and 0.6 W/cm^2^, indicating that the former produced more ^1^O_2_. In the presence of GSH, CM@OABACe6/Cu NPs have higher photosensitive properties, which is due to the ability of Cu^2+^ to deplete GSH. Under laser irradiation, the fluorescence strength of SOSG in OABACe6 NPs dispersion with GSH increased significantly, but the same concentration of GSH inhibited the fluorescence strength of SOSG in the OABACe6 NPs dispersion system to a certain extent. These results indicated that CM@OABACe6/Cu NPs could play a better PDT role than OABACe6 NPs in tumor environments. What is noteworthy is that the GSH depletion capacity of CM@OABACe6/Cu NPs is not only conducive to the effective generation of ^1^O_2_ but also conducive to the generation of ·OH through Fenton-like reactions, thus enhancing the ROS-related therapeutic effect. In conclusion, the multiple stimulation response characteristics of CM@OABACe6/Cu NPs enable them to realize the synergistic treatment of CDT and PDT in tumors under the guidance of fluorescence imaging.

In the field of biomedicine, one of the crucial characteristics of nanomedicine is good stability. CM@OABACe6/Cu NPs were incubated in RPMI 1640 and PBS (pH 7.4), respectively, and their stability was investigated through particle size changes (Fig. 4g, h). Stability of OABACe6/Cu NPs in PBS (pH 7.4) and RPMI 1640 (Fig. S6). In addition, SEM imaging of samples collected from days 1, 4 and 7 showed that the morphology of the NP remained unchanged (Fig. S7). Furthermore, SEM imaging of samples collected from days 1, 4 and 7 showed that the morphology of the NP remained unchanged (Fig. S7). Meanwhile, TEM imaging of samples taken from days 1, 4 and 7 showed that the cell membrane was still wrapped around the surface of the NPs (Fig. S8). The results show that the OABACe6 NPs and CM@OABACe6/Cu NPs show good stability. In addition, the Ce6 release of CM@OABACe6/Cu NPs in different media was monitored (Fig. 4i). The release rates of free Ce6 at pH 7.4 and 6.5 were 84.1% and 90.5% at 4 h, respectively, demonstrating rapid release. After 24 h, the release of Ce6 in CM@OABACe6/Cu NPs was 44.9% and 52.7% where the pH value was 7.4 and 6.5, respectively. At 48 h, only 5% (pH 7.4) and 6% (pH 6.5) of CM@OABACe6/Cu NPs were released, which further confirmed the stability of CM@OABACe6/Cu NPs. Similarly, Cu2 + can be effectively released in the tumour microenvironment. The CM@OABACe6/Cu NPs released 55.7% and 60.3% of Cu^2 +^ at 24 h and 48 h respectively (Fig. S9). It demonstrated that Cu^2 +^ can be effectively released in the tumour microenvironment. In addition, SEM imaging of samples collected at 0 h and 12 h of in vitro release showed that cleavage of NPs occurred at 12 h compared to 0 h, indicating that NPs were broken down in the tumour microenvironment (Fig. S10). Therefore, CM@OABACe6/Cu NPs have better stability and dispersity, can prolong blood circulation, and accumulate in tumor tissue, thereby ensuring the potential of PDT application in vivo.

### In Vitro FL Imaging and Synergistic Therapy of Chemo, CDT, and PDT

**Fig. 5:**
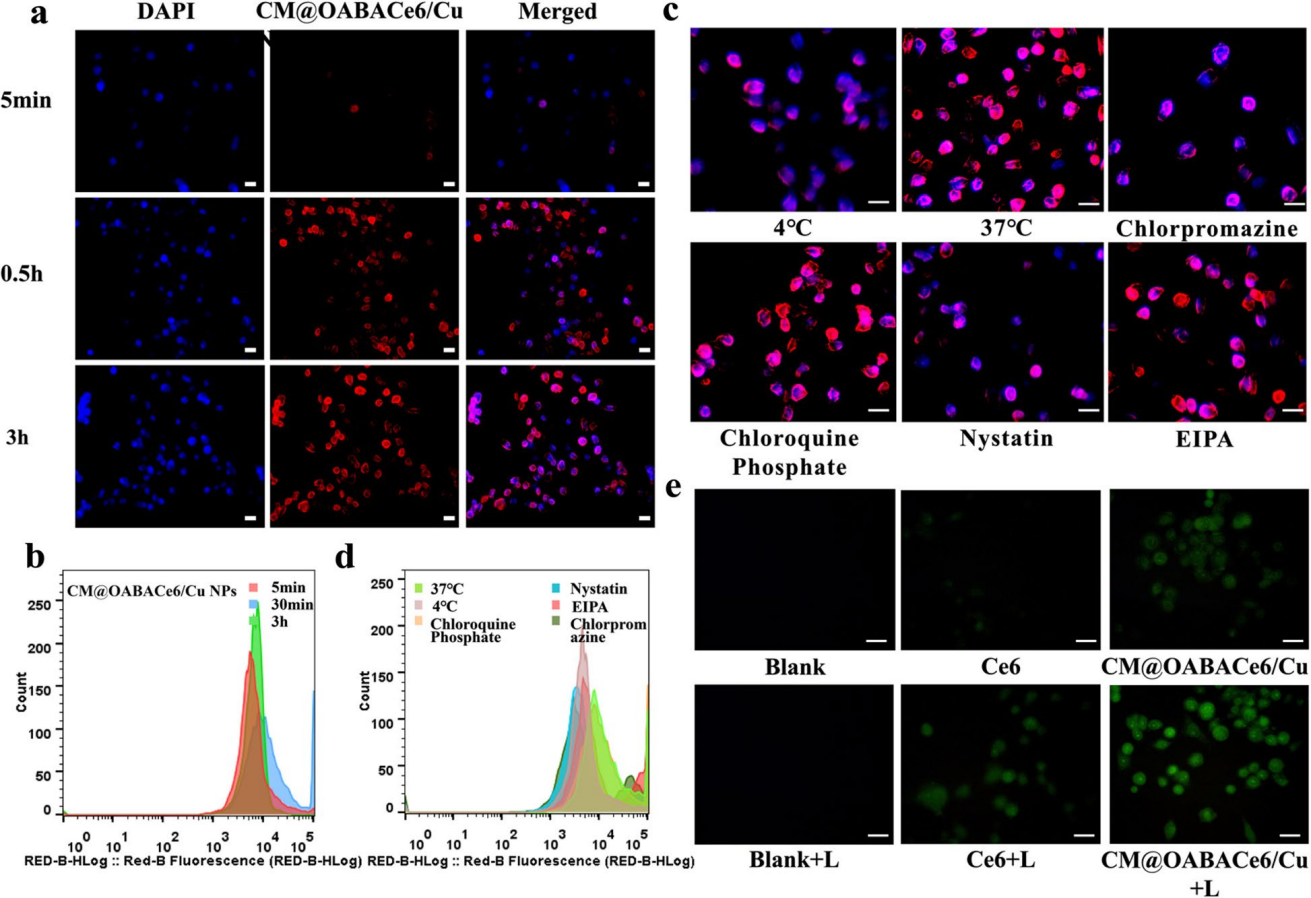
**a** FIM images of 4T1 cells treated with CM@OABACe6/Cu NPs at different times (blue for DAPI and red for NPs), scale bar: 15 μm, and **b**) corresponding flow cytometric analysis of fluorescence intensity. **c** FIM images of 4T1 cells treated with different endocytosis inhibitors (blue for DAPI and red for NPs), scale bar: 15 μm, and **d**) corresponding flow cytometric analysis of fluorescence intensity. **e** FIM images of 4T1 cells after treatment with DCFH-DA, followed by treatment with PBS (blank). PBS + laser, Ce6, Ce6 + laser, CM@OABACe6/Cu NPs, and CM@OABACe6/Cu NPs + laser, respectively (green for DCFH-DA), scale bar: 15 μm

Based on the unique TME response characteristics of CM@OABACe6/Cu NPs, the anticancer effects of CM@OABACe6/Cu NPs were studied in vitro at the cellular level. First, fluorescence inversion microscopy (FIM) and flow cytometry were used to monitor the time-dependent behavior of cell uptake of CM@OABACe6/Cu NPs. The results are shown in Fig. 5a. In the cell uptake experiment, the nanocarriers showed rapid uptake behavior. After incubation with OABACe6 NPs (Fig. S11, 12). or CM@OABACe6/Cu NPs for 30 min, obvious fluorescence signals could be found in the cytoplasm around the blue-stained 4T1 cell nucleus. Compared with the free Ce6 group (Fig. S13, 14), the fluorescence signal of OABACe6 NPs and CM@OABACe6/Cu NPs was significantly enhanced. After 3 h of incubation, the fluorescence was significantly enhanced. Based on the results, and cellular uptake showed a concentration–time dependence. This indicates that nanocarriers have a high affinity for cell membranes. The negatively charged particles bind to the cation site in cells, thereby increasing cell absorption of negatively charged nanocarrier. In addition, flow cytometry quantitative analysis results (Fig. 5b) showed that cells incubated with CM@OABACe6/Cu NPs (the mean fluorescence intensity of CM@OABACe6/Cu NPs was 21,102) had higher fluorescence strength compared with free Ce6 (the mean fluorescence intensity of free Ce6 was 10,322), especially after 3 h incubation, CM@OABACe6/Cu NPs showed significant fluorescence enhancement. The mean fluorescence intensity of each group of NPs is shown in Table S5. This enhanced uptake behavior may be attributed to the effective internalization of CM@OABACe6/Cu NPs through intracellular under complex microenvironment stimulation, and the subsequent decomposition of CM@OABACe6/Cu NPs under complex microenvironment stimulation and the release of partial Ce6. It has also been demonstrated that cell membranes have certain targeting properties, which increase their accumulation in tumor cells. In the meantime, free Ce6 diffuses poorly across the membrane. Similarly, we validated the targeting effect of non-cancerous cell lines in the L929 cell uptake assay. The results are shown in Figures S15-17 and Table S6. The uptake of CM@OABACe6/Cu NPs by L929 cells was significantly lower than that of 4T1 cells, indicating that CM@OABACe6/Cu NPs have superior tumour homing properties.

Subsequently, the effects of low temperature and endocytosis inhibitors on cell uptake were studied to evaluate cell internalization. Based on the results of the above cell uptake experiments, the fluorescence intensity was stronger after 3 h incubation. Therefore, we chose to add different inhibitors and incubate with the cells for 3 h to examine the pathway of NPs entry into the cells. The results showed (Fig. 5c) that the fluorescence intensity of 4T1 cells cultured at 37℃ was stronger than that of 4℃, which demonstrated that the uptake of 4T1 cells was mainly achieved through endocytosis. Chlorpromazine endocytosis inhibitors and Nystatin endocytosis inhibitors showed significantly lower uptake fluorescence intensity than Chloroquine Phosphate inhibitors and EIPA endocytosis inhibitors. These results indicated that the endocytosis of CM@OABACe6/Cu NPs is the result of multiple pathways, including caveolin-mediated endocytosis and clathrin-mediated endocytosis, and the whole process is energy-dependent. Meanwhile, flow cytometry quantitative analysis further supported the above conclusion (Fig. 5d).

Then, the intracellular ROS levels were further estimated using fluorescence 2,7-dichlorodiacetate (DCFH-DA) as a fluorescent indicator. As shown in Fig. 5e, no significant FL was observed in the blank group. The intercellular ROS production induced by CM@OABACe6/Cu NPs was higher than that induced by Ce6, which further indicated that CM@OABACe6/Cu NPs had better photodynamic effects. Notably, FL emission from cells treated with CM@OABACe6/Cu NPs became more intense under laser irradiation, suggesting that the combination of ·OH and ^1^O_2_ effectively increased ROS production.

**Fig. 6:**
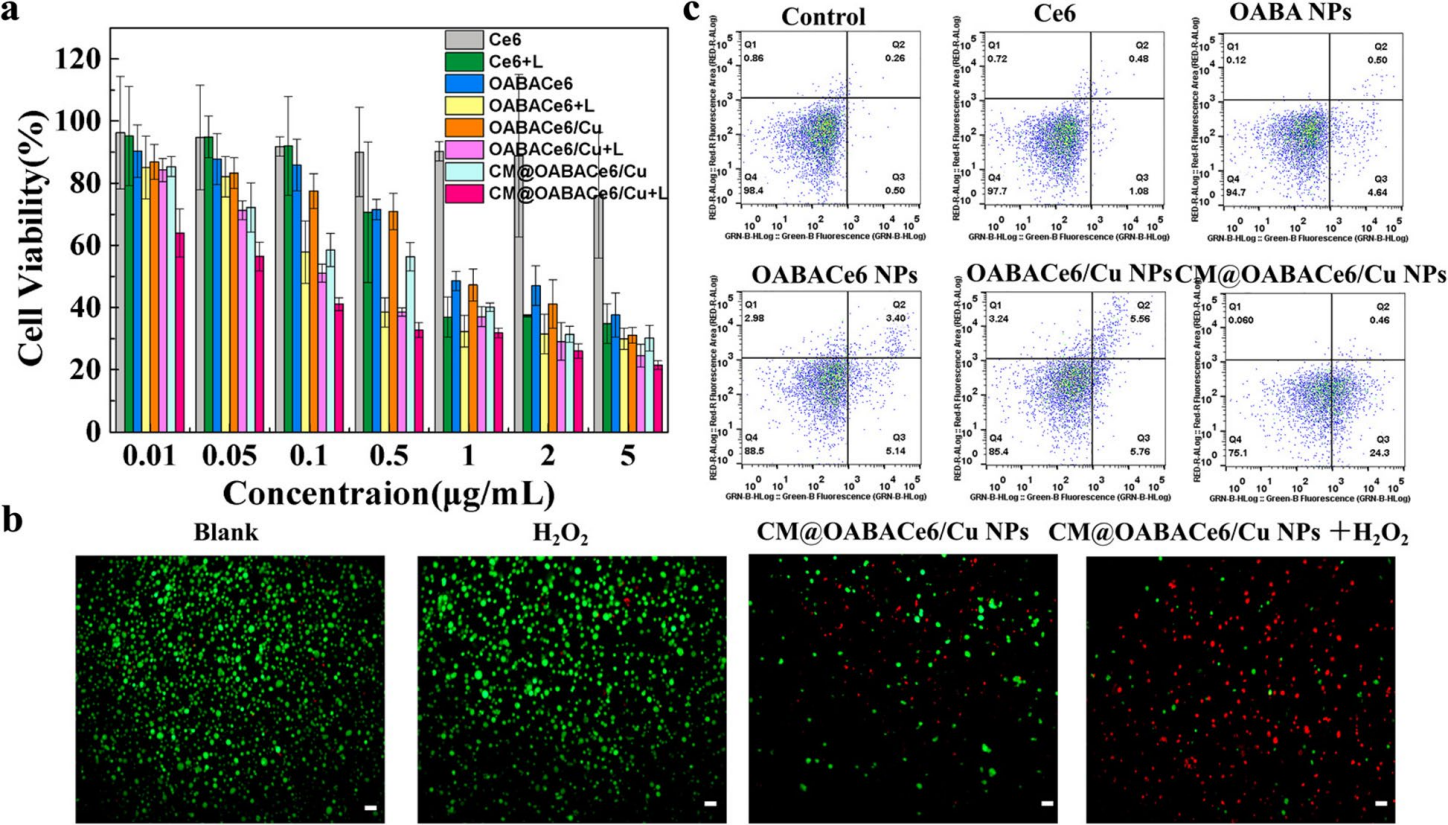
**a** Cell viability of 4T1 cells after treatment with various concentrations of CM@OABACe6/Cu NPs (from 0.01 to 5 μg/mL) and incubated at 37℃ for 24 h. **b** Calcein-AM/PI staining images of 4T1 cells after incubation with PBS (blank), H_2_O_2_, CM@OABACe6/Cu NPs, and the mixture of CM@OABACe6/Cu NPs, and H_2_O_2_, respectively, scale bar: 10 μm. (green for Calcein-AM and red for PI). **c** Apoptosis of 4T1 cells after incubation with PBS (control), Ce6, OABA NPs, OABACe6 NPs, OABACe6/Cu NPs, and CM@OABACe6/Cu NPs, respectively

To further evaluate the in vitro cytotoxicity of CM@OABACe6/Cu NPs and PDT effects, a standard methylthiazole-polyphenylterrazole-ammonium bromide (MTT) was used (Fig. 6a). The results showed that different concentrations of OABACe6 NPs and CM@OABACe6/Cu NPs could inhibit the proliferation of 4T1 cells. It could be seen that the survival rate of 4T1 cells decreased rapidly with the increase of equivalent Ce6 concentration. In other words, the survival rate of 4T1 cells was 41% and 31% when the equivalent Ce6 concentration in OABACe6 NPs and CM@OABACe6/Cu NPs was 2 μg/mL, respectively. The results showed that NPs coated with cancer cell membranes were more cytotoxic to 4T1 cells than uncoated ones. This is mainly due to the tumor-homing property of the cell membrane, which allows more nanocarriers to accumulate around the cells. The survival rate of irradiated NPs was significantly lower than that of non-irradiated nanocarriers. The survival rate of CM@OABACe6/Cu NPs was 26.1% when the equivalent Ce6 concentration was 2 μg/mL, indicating that CM@OABACe6/Cu NPs have better photodynamic effects. To explore the pharmacodynamic relationship of CM@OABACe6/Cu NPs, NPs combination index curves were drawn. Significantly, the IC50 of Ce6 in CM@OABACe6/Cu NPs (0.263 mg/mL) was much lower than that of free Ce6 (1.206 mg/mL), indicating a significant increase in PDT efficacy. Similarly, relatively low IC30 and IC70 values were observed in the co-assembly compared to the free Ce6 (Table S7). Meanwhile, CM@OABACe6/Cu NPs were more effective in combination chemotherapy/PDT/CDT on 4T1 cells compared to Ce6 PDT alone, showing a synergistic effect (combination index: CI < 1) (Figs. S18, 19). Furthermore, the specific CDT effect of CM@OABACe6/Cu NPs was further confirmed by Calcein-AM/PI staining images. The results showed the distribution of live (green) and dead (red) 4T1 cells in blank and CM@OABACe6/Cu NPs treated groups (Fig. 6b). Compared with the H_2_O_2_-treated group without apparent cell death, the majority of cells co-cultured in CM@OABACe6/Cu NPs mixed with H_2_O_2_ were killed, which verified the CDT triggered by H_2_O_2_. The OABACe6/Cu NPs group also showed the same experimental results (Fig. S20). Then, the effect of in vitro chemotherapy was assessed by observing the apoptosis induced by CM@OABACe6/Cu NPs (Fig. 6c). The early apoptosis rates were 1.05% and 4.84% in the Ce6 and OABA NPs groups, respectively. Since Ce6 was not biologically active without irradiation, it could not induce apoptosis in 4T1 cells, whereas OABA NPs had anti-tumor activity and it induced early apoptosis in 4T1 cells. Compared with OABACe6 NPs and OABACe6/Cu NPs groups, CM@OABACe6/Cu NPs group induced the highest number of early apoptosis (24.3%). It is certificated that CM@OABACe6/Cu NPs have an antitumor effect. In conclusion, CM@OABACe6/Cu NPs exhibit FL imaging in response to TME stimulation in vitro, as well as the synergistic anti-tumor properties of CDT, chemo, and PDT, and have good tumor targeting.

### In vivo biodistribution and tumor inhibition

**Fig. 7:**
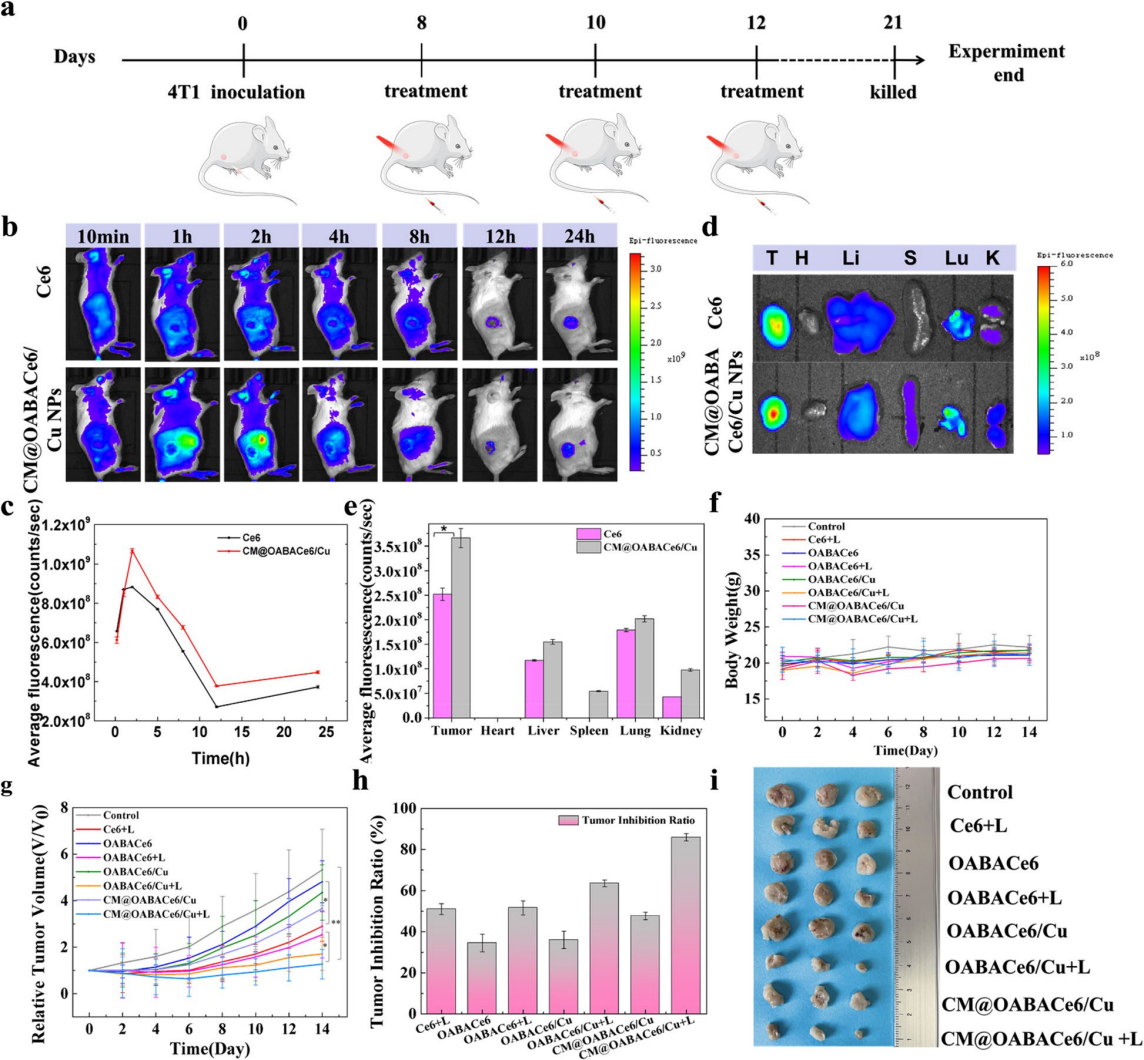
In vivo bio-distribution and cancer therapeutic efficacy of CM@OABACe6/Cu NPs. Imaging was performed at different time points after i.v. injection of CM@OABACe6/Cu NPs and free Ce6. **a** The overall timetable for animal experiments. **b** In vivo fluorescence imaging of 4T1 tumor-bearing mice, **c** Average fluorescence intensity of 4T1 tumor-bearing mice. **d** FL images of tumors and major organs (H: heart, Lu: lung, Li: liver, S: spleen, and K: kidneys) excised from mice after i.v., and **e**) average fluorescence intensity. **f** Changes in body weight of tumor-bearing mice in different treatment groups. **g** Tumor growth curves in different treatment groups during the monitoring period. **h**) Tumor suppression rates of mice in different treatment groups after 14 days (*p < 0.05 and **p < 0.01). **i** Photographs of dissected tumor tissues of each treatment group after 14 days

Encouraged by TME-triggered fluorescence imaging and the synergistic therapeutic properties of CM@OABACe6/Cu NPs at the cellular level, their biological distribution in vivo and antitumor effects have been further investigated. First, the hemocompatibility of CM@OABACe6/Cu NPs was assessed by hemolysis assay to determine their safety in antitumor applications. As shown in Fig. S21, no significant erythrocyte color was observed for different concentrations of CM@OABACe6/Cu NPs compared to the control group, indicating the high blood compatibility of the NPs. The biodistribution of CM@OABACe6/Cu NPs at tumor sites in mice was evaluated and measured by an animal fluorescence imaging system (Fig. 7b, c). CM@OABACe6/Cu NPs accumulated rapidly in tumors after intravenous injection, and the highest level of FL signal was detected in tumors 2 h after injection, which may be due to prolonged blood circulation and Active targeting effects were enhanced. Until the end of observation (24 h), there was still weak fluorescence emission from the tumor site, which verified the effective accumulation of nano-components at the tumor site. In vitro imaging of major organs and tumors further demonstrated excellent tumor-targeting ability (Fig. 7d, e). In tumour, cancer cell surface antigens have homologous or heterologous adhesion properties, and NPs coated with cancer cell membrane (CCM) can compete with homologous cancer cell surface antigens to gain immune escape and homologous targeting capabilities for highly specific cancer targeting and effective cancer therapy [42]. The liver and kidney also exhibit obvious FL signals, which indicated that CM@OABACe6/Cu NPs could be removed from mice through the kidney and liver pathways. Notably, Ce6 also showed a lower selective accumulation on tumors [44], and its fluorescence intensity is weaker than that of CM@OABACe6/Cu NPs. The fluorescence distribution of the OABACe6 NPs and OABACe6/Cu NPs groups and the fluorescence intensity of each tissue organ are shown in Figs. S22-25.

Encouraged by the superior tumor-targeting capabilities and nano assemblies that have synergistic therapeutic effects, we systematically investigated the injected doses using 4T1 tumor-bearing mice. Different doses of CM@OABACe6/Cu NPs were administered (Ce6 equivalent: 2.5 mg/kg, 3.5 mg/kg, and 4.5 mg/kg). The drug was administered on day 0 and then irradiated for 15 min 6 h after injection (675 ± 10 nm light, 150 mW/cm^2^). The results showed that the tumor inhibition rate of Ce6 equivalent to 4.5 mg/kg was 71.8%, which was significantly higher than Ce6 equivalent to 3.5 mg/kg (53.8%) and Ce6 equivalent to 2.5 mg/kg (41.9%) (Fig. S26-28). Therefore, Ce6 equivalent to 4.5 mg/kg was selected as the optimal administration dose for subsequent tumor therapy.

Subsequently, the tumor-bearing mice were randomly divided into eight groups: 1) Blank group (PBS), 2) Control group (Ce6 + Laser), 3) Chemo group (OABACe6 NPs), 4) Chemo + PDT group (OABACe6 NPs + Laser), 5) Chemo + CDT group (OABACe6/Cu NPs), 6) Chemo + CDT + PDT group (OABACe6/Cu NPs + laser), 7) Targeted + Chemo + CDT group (CM@OABACe6/Cu NPs), and 8) Targeted + Chemo + CDT + PDT group (CM@OABACe6/Cu NPs + laser). Mice were injected intravenously on days 0, 2, and 4, followed by 15 min of irradiation 6 h after each injection. Figure 7a shows the general schedule of animal experiments. After treatment, tumor volume and weight of mice were recorded regularly (Fig. 7f, g). The results showed that OABACe6 NPs, OABACe6/Cu NPs, and CM@OABACe6/Cu NPs groups all showed a certain growth inhibition effect compared with the control group (Fig. 7h). The tumor inhibition rate of the CM@OABACe6/Cu NPs group was 47.7%, which was higher than that of the OABACe6 NPs group (36.0%) and OABACe6/Cu NPs group (34.4%). Owing to Chemo and CDT effects of CM@OABACe6/Cu NPs and certain targeting effects, mice in the Targeted + Chemo + CDT group showed obvious antitumor effects. Meanwhile, the Chemo + PDT group and Chemo + CDT + PDT group showed significant inhibition of tumor growth after laser irradiation, with tumor inhibition rates of 51.6% and 63.4%, respectively, indicating that Chemo and CDT combined with PDT had significant anti-tumor effects. Unsurprisingly, the highest tumor suppression rate (85.9%) was observed in the Target + Chemo + CDT + PDT group. The highest efficacy of the combination therapy was validated. Tumor autopsy photos 14 days after treatment showed that the tumors of CM@OABACe6/Cu NPs phototherapy mice were also smaller than those of the other groups (Fig. 7i). The weights of tumours and various tissues and organs in mice are shown in Fig. S29, 30.

**Fig. 8:**
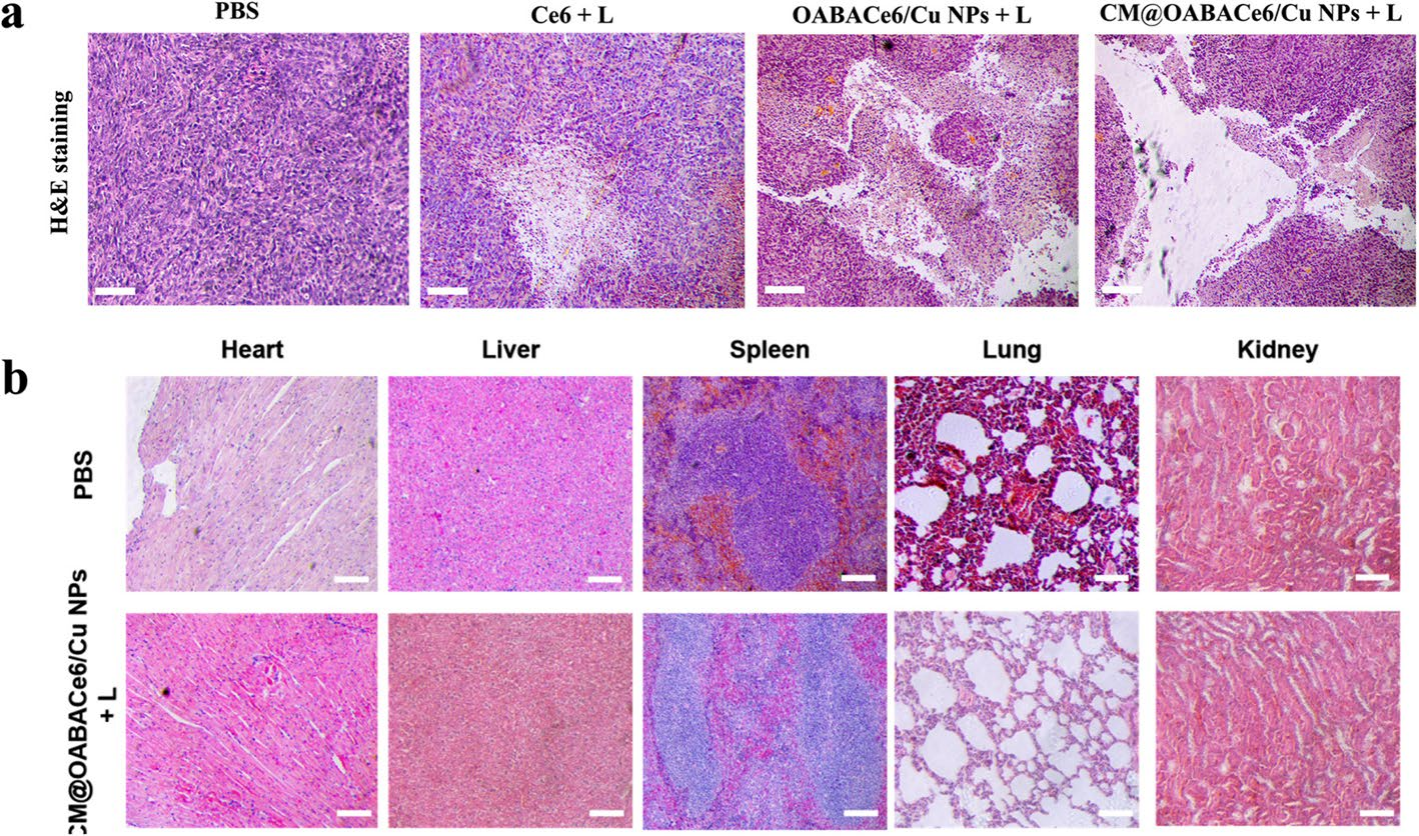
**a** H&E staining of the tumor sections of mice after various treatments as indicated for 14 days, scale bar: 20 μm. H&E staining of major organs gathered from representative mice of PBS and CM@OABACe6/Cu NPs + L groups, scale bar: 20 μm

H&E staining was done on different groups of tumors at the end of the monitoring period to elucidate the corresponding tumor damage effects of different treatments (Fig. 8a). H&E results showed that CM@OABACe6/Cu NPs group showed obvious tissue necrosis after irradiation, which further confirmed that CM@OABACe6/Cu NPs had a good therapeutic effect on PDT. Furthermore, the in vivo toxicity of CM@OABACe6/Cu NPs was evaluated by observing the weight change of mice. During the 14-day treatment, although the weight of mice decreased slightly in the first few days, it gradually returned to a normal level in the later period of observation. After 14 days of treatment, major organs including the heart, liver, spleen, lung, and kidney were collected for histological analysis (Fig. 8b). The H&E-stained results indicated that no obvious pathological change or other adverse changes can be found in all of the organs. In conclusion, CM@OABACe6/Cu NPs have better biocompatibility and biodegradability, low toxicity in vivo, and it has great synergistic antitumor potential as a promising bionic targeted drug carrier.

## Discussion

In this study, we designed CM@OABACe6/Cu NPs with anti-tumour properties and tumour targeting, a multifunctional nano-combination with the following advantages. Firstly, the triterpenes as chemotherapeutic agents have some anti-tumour activity, which can improve the therapeutic effect. Secondly, the Cu^2 +^ in the nano-combination not only depletes excess GSH in the cells, but also reacts with endogenous hydrogen peroxide to produce highly toxic ·OH [35–37], making it possible to become CDT. Third, nanocomposites encapsulated within 4T1 can target tumour sites due to the homing properties of 4T1 cells [40]. Finally, the combination of Ce6, Cu^2 +^ and triterpenes gives the nanocomposite a synergistic effect of PDT, CDT and chemotherapy. The constructed nano-loaded platform increased the accumulation of the composite nanomaterials at the tumour site by homologously targeting the cancer cell membrane and improved the efficacy of the tumour treatment by combined chemotherapy-PDT-CDT treatment.

To investigate the mechanism of carrier-drug interactions, molecular dynamics (MD) simulations were performed on OABACe6 NPs. The results showed that these molecules were constructed into NPs through hydrogen bonding forces as well as π-π interactions. Subsequently, Cu^2 +^ was introduced to endow the OABACe6/CuNPs with CDT capacity and exert GSH depletion in the tumour. The 4T1 cell membrane was then wrapped around the surface of the NPs to allow effective targeting of homologous tumours in vivo. The CM@OABACe6/Cu NPs showed rapid uptake by the cells compared to the other groups of NPs. This suggests that the nanoparticles have a higher affinity for the cell membrane. The cytotoxicity of NPs encapsulating cancer cell membranes was higher for 4T1 cells than for unencapsulated cells. This is mainly due to the tumour-homing properties of the cell membrane, which causes more nanoparticles to accumulate around the cells. The survival rate of the irradiated nanoparticles was significantly lower than that of the unirradiated nanoparticles, indicating that CM@OABACe6/Cu nanoparticles have better photodynamic effects.

In tumours, cancer cell surface antigens have homologous or heterologous adhesion properties, while NPs encapsulated by cancer cell membranes (CCM) can compete with homologous cancer cell surface antigens to gain immune escape and homologous targeting capabilities for highly specific cancer targeting and effective cancer therapy [36]. Encouraged by TME-triggered fluorescence imaging and the synergistic therapeutic properties of CM@OABACe6/Cu NPs at the cellular level, their biological distribution and anti-tumour effects in vivo have been further investigated. In vivo distribution experiments showed fluorescence emission at tumour sites in mice, which validated the effective accumulation of CM@OABACe6/Cu NPs at tumour sites. In vitro imaging of major organs and tumours further demonstrated good tumour targeting capabilities. Meanwhile, the Target + Chemo + CDT + PDT group showed the highest tumour inhibition rate (85.9%). The highest efficacy of the combination therapy was validated. However, MOA (mode of action) is an important part of understanding the mechanisms of tumour therapy. In this paper we have focused on the delivery and therapeutic effects of DDS in vivo and have not studied MOAof drugs. we will do these experiments in the future and report the results.

## Conclusions

In conclusion, we developed and validated a nano-drug delivery platform consisting of NSMs co-assembled with photosensitizers and introduced into Cu^2+^ and tumor cell membranes. A key feature of this platform is that it offers tumor homing targeting, triple synergistic therapies with chemotherapy, PDT, and CDT, as well as TME stimulation responsiveness. Triterpenoids such as these possess good antitumor activity, as well as the ability to co-assemble with Ce6 to achieve the synergistic antitumor activity. The tumor cell membrane endows the nanocarriers with good tumor targeting. The introduction of Cu^2+^ will not only provide CDT function but also effectively depletes GSH in the tumor, thus improving ROS-related therapeutic efficiency. Further, the nano-drug delivery platform has the advantages of biocompatibility and biosafety, minimal side effects, enhanced accumulation capacity at tumor sites, and significant anti-tumor activity. All in all, this study broadens the horizon of multi-component NSMs co-combined for the synergistic treatment of cancer and extends the use of bioactive NSMs in medicine.

## Supplementary Information

**Additional file 1: Figure S1.** SEM images of natural small moleculeco-assembled NPs. **Figure S2.** The cell viability of 4T1 cells incubated with different concentrations of co-assembled nanoparticles for 24 hours was evaluated. **Table S1.** OAUACe6 NPs particle size and PDI (OA:UA:Ce6,v:v:v ). **Table S2.** Ce6 loading content of NPs. **Table S3.** Ce6 loading content of OABACe6. **Table S4.** NPs particle size and PDI. **Figure S3.** Molecular skeleton configuration of OABA NPs, and OABACe6 NPs. **Figure S4.** SEM image of OABACe6/Cu NPs. **Figure S5.** Protein expression of 4T1 cell membrane and CM@OABACe6/CuNPs. **Figure S6.** OABACe6 NPs were incubated in RPMI 1640 (containing 10% FBS) and PBS (pH 7.4), respectively, and their stability was investigated by the particle size change. **Figure S7.** SEM image of CM@OABACe6/Cu NPs in PBS (pH 7.4). **Figure S8.** TEM image of CM@OABACe6/Cu NPs at PBS (pH 7.4). **Figure S9.** In vitro release of CM@OABACe6/Cu NPs in pH 6.5 release media (GSH 200 μM). **Figure S10.** SEM imaging of samples collected at 0 h and 12h from in vitro release (pH 6.5). **Figure S11. **OABACe6 NPs (equivalent Ce6 concentration of 2 μg/mL) and 4T1 cells were incubated for 5 min, 30 min, and 3 h, respectively. A fluorescence inverted microscope was used for imaging, scale bar: 15 μm (bluefor DAPI and red for NPs). **Figure S12.** OABACe6 NPs (equivalent Ce6 concentration of 2 μg/mL) and 4T1 cells were incubated for 5 min, 30 min, and 3 h, respectively. The quantitative analysis was performed by flow cytometry. **Figure S13.** Free Ce6 (equivalent Ce6 concentration of 2 μg/mL) and 4T1 cells were incubated for 5 min, 30 min, and 3 h, respectively. A fluorescence inverted microscope was used for imaging, scale bar: 15 μm (blue for DAPI and red for NPs). **Figure S14.** Free Ce6 (equivalent Ce6 concentration of 2 μg/mL) and 4T1 cells were incubated for 5 min, 30 min, and 3 h, respectively. The quantitative analysis was performed by flow cytometry. **Table S5.** The mean fluorescence intensity of NPs. **Figure S16.** CM@OABACe6/Cu NPs (equivalent Ce6 concentration of 2 μg/mL) and L929 cells were incubated for 5 min, 30 min, and 3 h, respectively. A fluorescence inverted microscope was used for imaging, scalebar: 15 μm (blue for DAPI and red for NPs). **Figure S17.** Free Ce6 and CM@OABACe6/Cu NPs (equivalent Ce6 concentration of 2 μg/mL) and L929 cells were incubated for 5 min, 30 min, and 3 h, respectively. The quantitative analysis was performed by flow cytometry. **Table S6.** The mean fluorescence intensity of NPs. **Table S7.** The IC30, IC50, IC70 analysis of free Ce6 and CM@OABACe6/Cu NPs against 4T1, respectively. **Figure S18.** Combination index(CI) of CM@OABACe6/Cu NPs on 4T1 cells, as determined by ChouTalalay theorem calculation. **Figure S19.** Combination index(CI) of OABACe6/Cu NPs and OABACe6NPs on 4T1 cells, as determined by ChouTalalay theorem calculation. **Figure S20.** Calcein-AM/PI staining images of 4T1 cells after incubation with OABACe6/Cu NPs, and the mixture of OABACe6/Cu NPs, and H_2_O_2_, respectively, scale bar: 10 μm. (green for Calcein-AM and red for PI). **Figure S21.** Photograph of hemolytic assay result ofcontrols, OABACe6 NPs, and CM@OABACe6/Cu NPs at different concentrations (100, 200, and 400 μg/mL). **Figure S22.** Imaging was performed at different time points after i.v. injection of OABACe6 NPs and OABACe6/Cu NPs. In vivo fluorescence imaging of 4T1 tumor-bearing. **Figure S23.** Imaging was performed at different time points after i.v. injection of OABACe6 NPs and OABACe6/Cu NPs. Average fluorescence intensity of 4T1 tumor-bearing mice. **Figure S24.** Imaging was performed at different time points after i.v. injection of OABACe6 NPs and OABACe6/Cu NPs. FL images of tumors and major organs (H: heart, Lu: lung, Li: liver, S: spleen, and K: kidneys) excised from mice after i.v. **Figure S25.** Imaging was performed at different time points after i.v. injection of OABACe6 NPs and OABACe6/Cu NPs. Mean fluorescence intensity of tumors and major organs (H: heart, Lu: lung, Li: liver, S: spleen, and K: kidneys) excised from mice after i.v. **Figure S26.** *In vivo,* systematic studies focus on the optimization of dosing. The tumor inhibition rate after 12 days of PDT treatment at different doses (Ce6 equivalent: 2.5 mg/kg, 3.5 mg/kg, 4.5 mg/kg). **Figure S27.*** In vivo*, systematic studies focus on the optimization of dosing. Relative tumor volume after 12 days of PDT treatment at different doses (Ce6 equivalent: 2.5 mg/kg, 3.5 mg/kg, 4.5 mg/kg).(*p<0.05 and **p<0.01, Student’s t test). **Figure S28.** *In vivo*, systematic studies focus on the optimization of dosing. Weight changes were observed during 12 days of PDT treatment with different doses (Ce6 equivalent: 2.5 mg/kg, 3.5 mg/kg, 4.5 mg/kg). **Figure S29. **Tumor weight in the eight treatment groups at the end of treatment. **Figure S30. **The weight of each organ in the eight treatment groups after treatment.

## Data Availability

Not applicable.
