# Supplementary material for: Bionic natural small molecule co-assemblies towards targeted and synergistic Chemo/PDT/CDT
Source: Biomater Res. 2023 May 9;27:43. doi: 10.1186/s40824-023-00380-z (PMC10169343; doi:10.1186/s40824-023-00380-z)
Supplement: Supplementary file 1 — Additional file 1: Figure S1. SEM images of natural small moleculeco-assembled NPs. Figure S2. The cell viability of 4T1 cells incubated with different concentrations of co-assembled nanoparticles for 24 hours was evaluated. Table S1. OAUACe6 NPs particle size and PDI (OA:UA:Ce6,v:v:v ). Table S2. Ce6 loading content of NPs. Table S3. Ce6 loading content of OABACe6. Table S4. NPs particle size and PDI. Figure S3. Molecular skeleton configuration of OABA NPs, and OABACe6 NPs. Figure S4. SEM image of OABACe6/Cu NPs. Figure S5. Protein expression of 4T1 cell membrane and CM@OABACe6/CuNPs. Figure S6. OABACe6 NPs were incubated in RPMI 1640 (containing 10% FBS) and PBS (pH 7.4), respectively, and their stability was investigated by the particle size change. Figure S7. SEM image of CM@OABACe6/Cu NPs in PBS (pH 7.4). Figure S8. TEM image of CM@OABACe6/Cu NPs at PBS (pH 7.4). Figure S9. In vitro release of CM@OABACe6/Cu NPs in pH 6.5 release media (GSH 200 μM). Figure S10. SEM imaging of samples collected at 0 h and 12h from in vitro release (pH 6.5). Figure S11. OABACe6 NPs (equivalent Ce6 concentration of 2 μg/mL) and 4T1 cells were incubated for 5 min, 30 min, and 3 h, respectively. A fluorescence inverted microscope was used for imaging, scale bar: 15 μm (bluefor DAPI and red for NPs). Figure S12. OABACe6 NPs (equivalent Ce6 concentration of 2 μg/mL) and 4T1 cells were incubated for 5 min, 30 min, and 3 h, respectively. The quantitative analysis was performed by flow cytometry. Figure S13. Free Ce6 (equivalent Ce6 concentration of 2 μg/mL) and 4T1 cells were incubated for 5 min, 30 min, and 3 h, respectively. A fluorescence inverted microscope was used for imaging, scale bar: 15 μm (blue for DAPI and red for NPs). Figure S14. Free Ce6 (equivalent Ce6 concentration of 2 μg/mL) and 4T1 cells were incubated for 5 min, 30 min, and 3 h, respectively. The quantitative analysis was performed by flow cytometry. Table S5. The mean fluorescence intensity of NPs. Figure S16. CM@OA [file 40824_2023_380_MOESM1_ESM.docx]

**Title Page**

**Bionic natural small molecule co-assemblies towards targeted and synergistic Chemo/PDT/CDT**

Shiyao Fu^1, 2, #^, Mingao Wang^3, #^, Bin Li^4, #^, Xu Li^5^, Jianjun Cheng^2^, Haitian Zhao^1, 6^, Hua Zhang^2^, Aijun Dong^2^, Weihong Lu^1, 6^ and Xin Yang^1, 2, 6, *^

*^1^ School of Medicine and Health, Harbin Institute of Technology, No. 92, West Dazhi Street, Nangang District, Harbin, 150001, China.*

*^2^ School of Chemistry and Chemical Engineering, Harbin Institute of Technology, No.92, West Dazhi Street, Nangang District, Harbin, 150001, China.*

*^3^ Department of Nephrology, the First Affiliated Hospital of Harbin Medical University, No. 23 Youzheng Street, Nangang District, Harbin, 150001, China.*

*^4^ Academician Workstation, Jiangxi University of Traditional Chinese Medicine, No. 1088 Meiling Street, Wanli District, Nanchang, 330004, China.*

*^5^ Department of Ophthalmology, the Second Hospital of Jilin University, No. 4026 Yatai street, Nanguan District, Changchun, 130041, China.*

*^6^ Chongqing Research Institute, Harbin Institute of Technology, No. 188 Jihuayuan South Road, Yubei District, Chongqing, 401135, China.*

**E-mail address:** yangxin@hit.edu.cn (**Corresponding authors** Xin Yang)

**Supporting Information**

**Supporting Figures:**


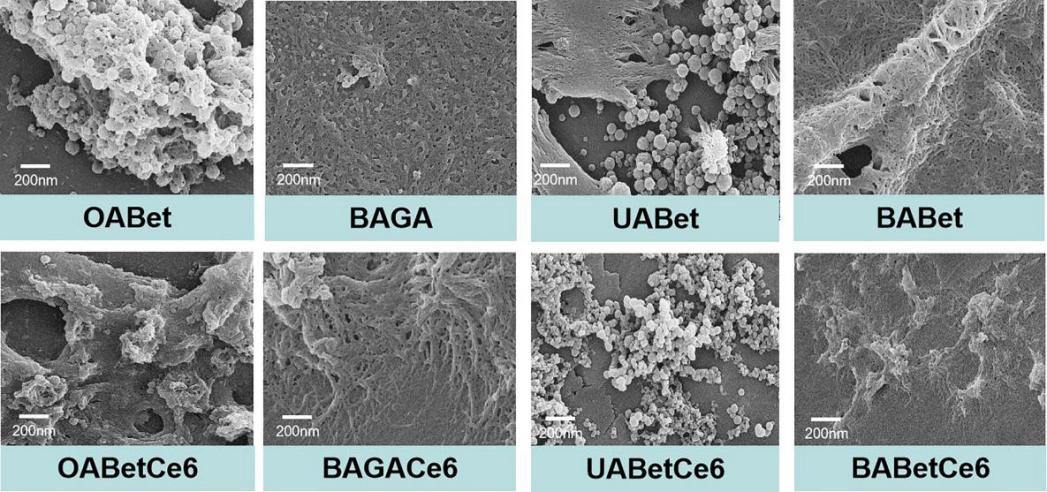


**Figure S1.** SEM images of natural small molecule co-assembled NPs.

**Figure S2.** The cell viability of 4T1 cells incubated with different concentrations of co-assembled nanoparticles for 24 hours was evaluated.

**Table S1.** OAUACe6 NPs particle size and PDI (OA:UA:Ce6, v:v:v ).

| **Nanoparticles** | **Diameter(nm)** | **PDI** |
| --- | --- | --- |
| 1:1:2 | 284.9 | 0.355 |
| 1:1:1 | 199.1 | 0.144 |
| 2:2:1 | 521.5 | 0.264 |

**Table S2.** Ce6 loading content of NPs.

| **NPs** | **Component** | **Concentration (μg/mL)** | **Drug loading (%)** |
| --- | --- | --- | --- |
| OABACe6 NPs | Ce6 | 32.81 | 3.28 |
| OAUACe6 NPs | Ce6 | 33.03 | 3.3 |
| OAGACe6 NPs | Ce6 | 26.55 | 2.15 |
| UABACe6 NPs | Ce6 | 51.68 | 5.17 |
| UAGACe6 NPs | Ce6 | 10.12 | 2.53 |

**Table S3.** Ce6 loading content of OABACe6.

| **Component** | **Concentration (μg/mL)** | **Drug loading (%)** |
| --- | --- | --- |
| OA | 540.3 | 54.03 |
| BA | 431.28 | 43.13 |
| Ce6 | 32.81 | 3.28 |

**Table S4.** NPs particle size and PDI.

| **Nanoparticles** | **Diameter(nm)** | **PDI** |
| --- | --- | --- |
| OABA | 133.43 ± 1.1 | 0.122±0.026 |
| OABACe6 | 146.6 ± 2.1 | 0.143±0.009 |
| OABACe6/Cu | 152.9 ± 1.5 | 0.159±0.006 |
| CM@OABACe6/Cu | 175.1 ± 1.1 | 0.122±0.001 |


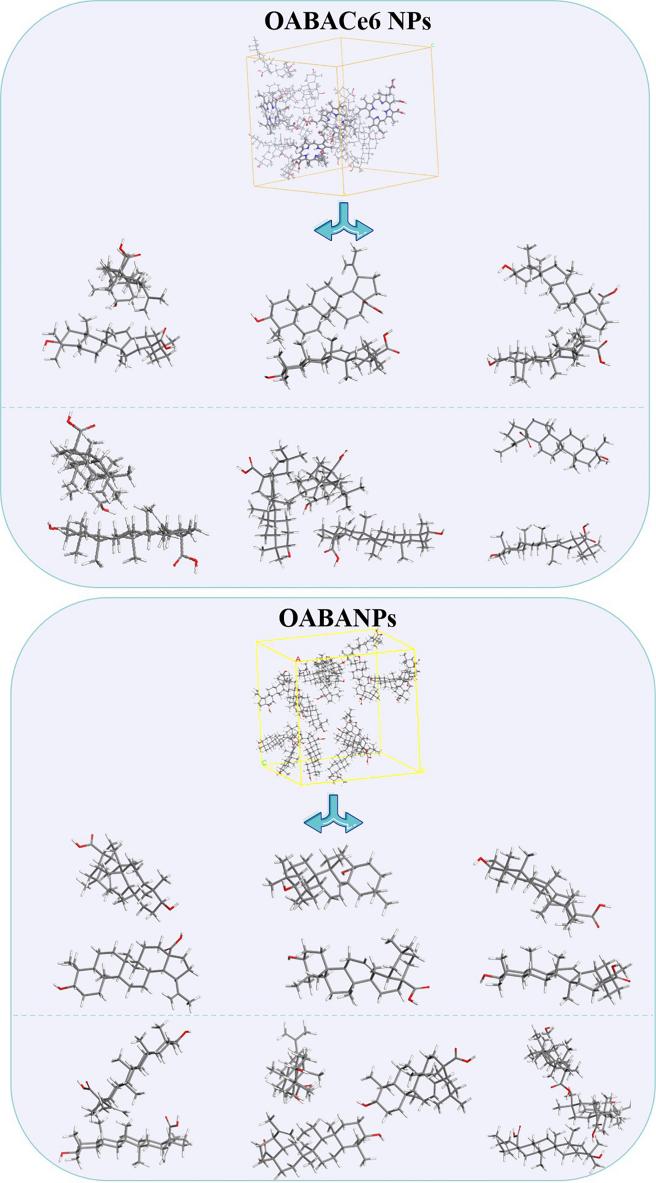


**Figure S3.** Molecular skeleton configuration of OABA NPs, and OABACe6 NPs.

**
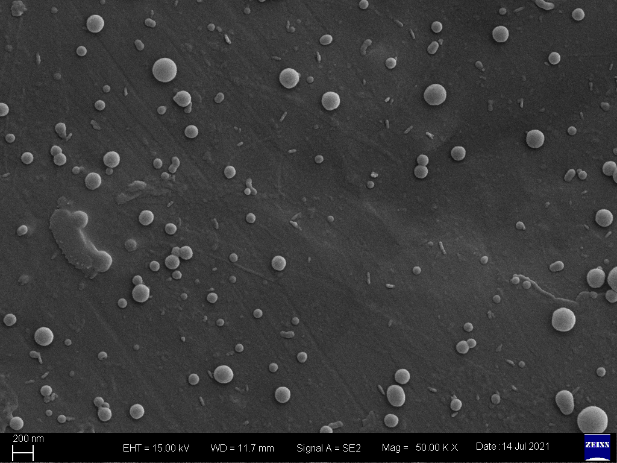
**

**Figure S4.** SEM image of OABACe6/Cu NPs.


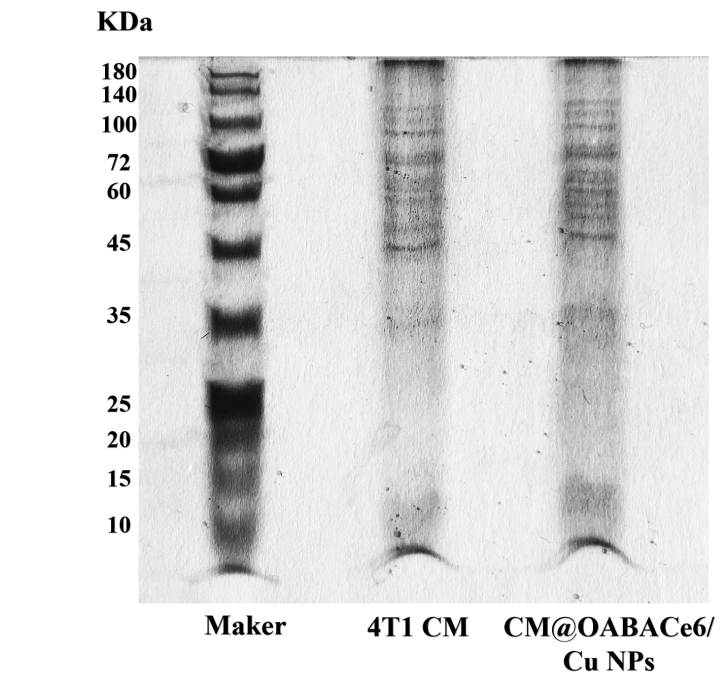


**Figure S5.** Protein expression of 4T1 cell membrane and CM@OABACe6/Cu NPs.

**Figure S6.** OABACe6 NPs were incubated in RPMI 1640 (containing 10% FBS) and PBS (pH 7.4), respectively, and their stability was investigated by the particle size change.


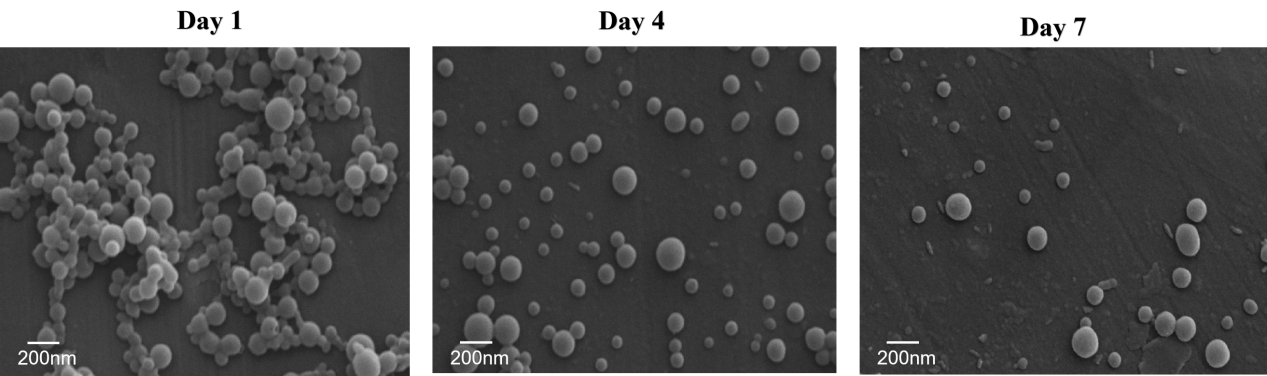


**Figure S7.** SEM image of CM@OABACe6/Cu NPs in PBS (pH 7.4).


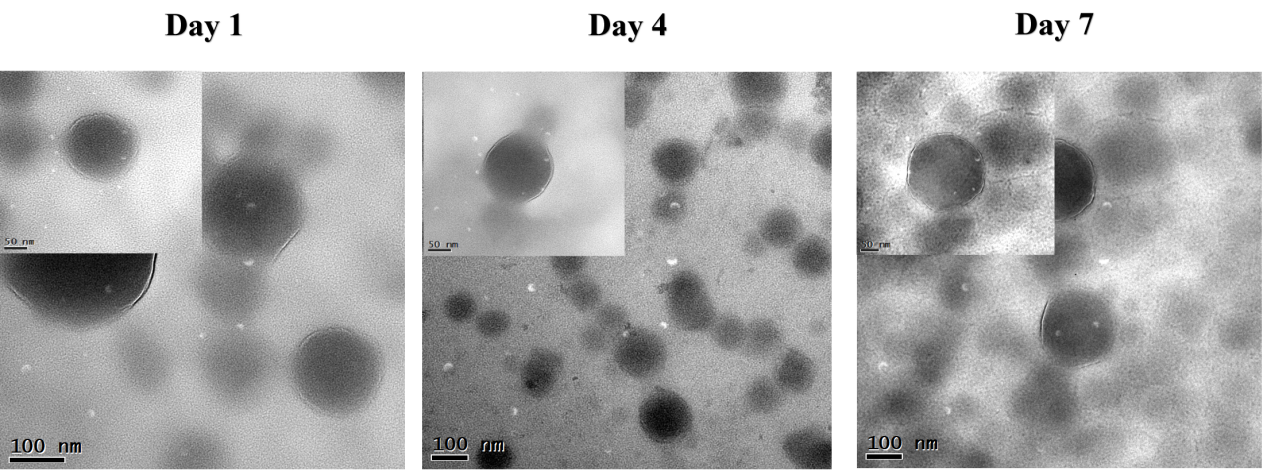


**Figure S8.** TEM image of CM@OABACe6/Cu NPs at PBS (pH 7.4).

**Figure S9.** In vitro release of CM@OABACe6/Cu NPs in pH 6.5 release media (GSH 200 μM).


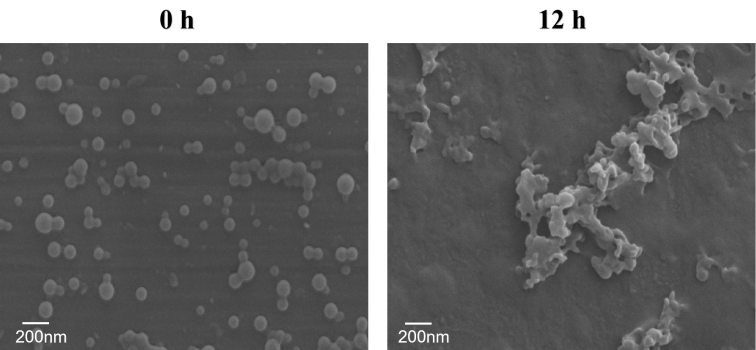


**Figure S10.** SEM imaging of samples collected at 0 h and 12 h from in vitro release (pH 6.5).


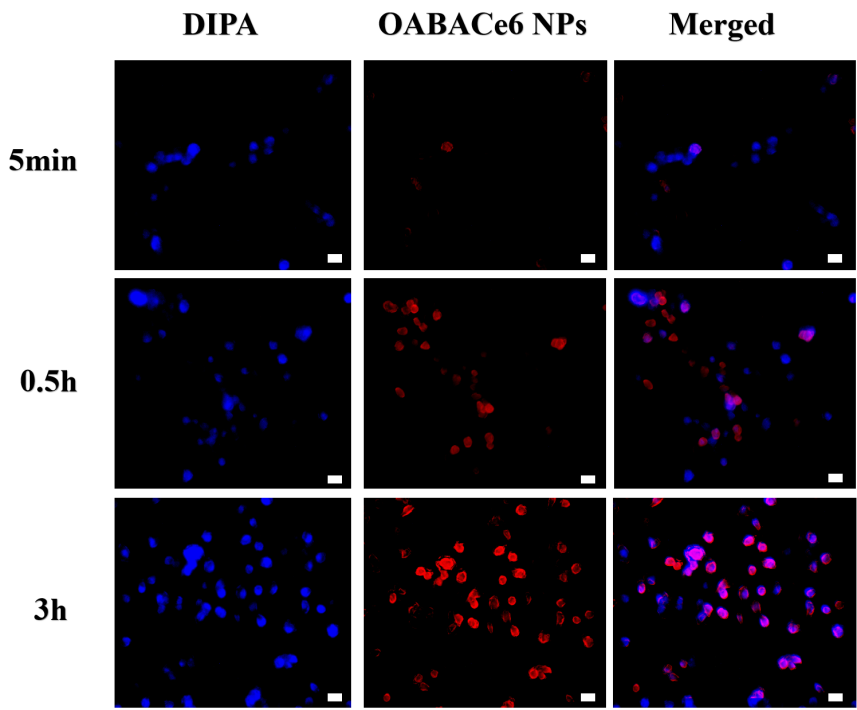


**Figure S11.** OABACe6 NPs (equivalent Ce6 concentration of 2 μg/mL) and 4T1 cells were incubated for 5 min, 30 min, and 3 h, respectively. A fluorescence inverted microscope was used for imaging, scale bar: 15 μm (blue for DAPI and red for NPs).


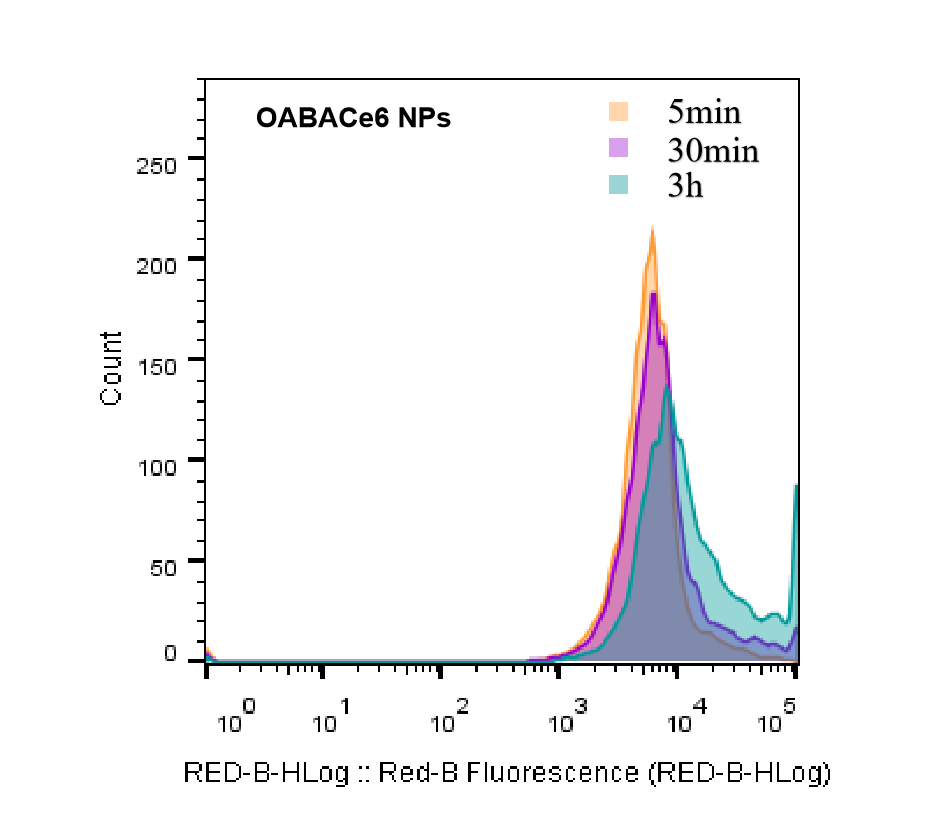


**Figure S12.** OABACe6 NPs (equivalent Ce6 concentration of 2 μg/mL) and 4T1 cells were incubated for 5 min, 30 min, and 3 h, respectively. The quantitative analysis was performed by flow cytometry.


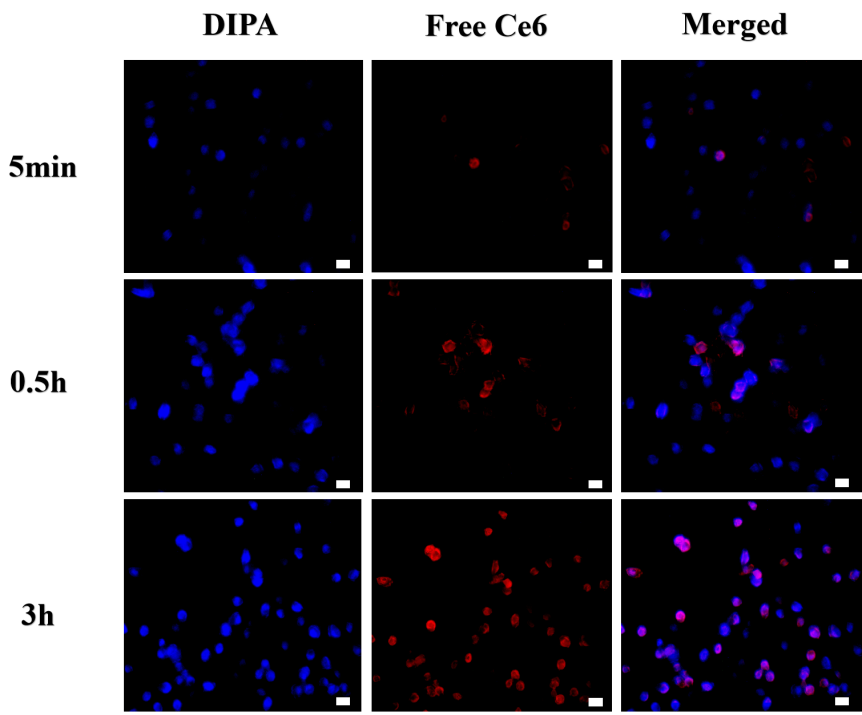


**Figure S13.** Free Ce6 (equivalent Ce6 concentration of 2 μg/mL) and 4T1 cells were incubated for 5 min, 30 min, and 3 h, respectively. A fluorescence inverted microscope was used for imaging, scale bar: 15 μm (blue for DAPI and red for NPs).


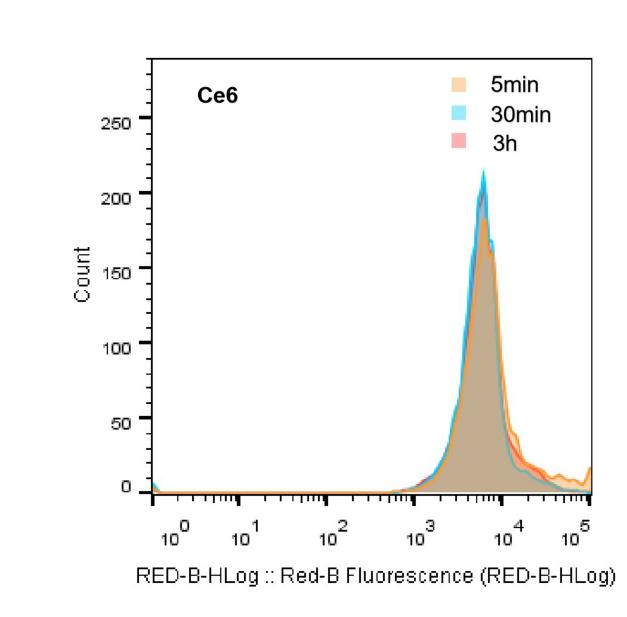


**Figure S14.** Free Ce6 (equivalent Ce6 concentration of 2 μg/mL) and 4T1 cells were incubated for 5 min, 30 min, and 3 h, respectively. The quantitative analysis was performed by flow cytometry.

**Table S5.** The mean fluorescence intensity of NPs.

| **Time** | **Nanoparticles** | | |
| --- | --- | --- | --- |
|  | Free Ce6 | OABACe6 NPs | CM@OABACe6/Cu NPs |
| 5min | 6986 | 6032 | 7411 |
| 0.5h | 6949 | 6196 | 8327 |
| 3h | 10322 | 18989 | 21102 |


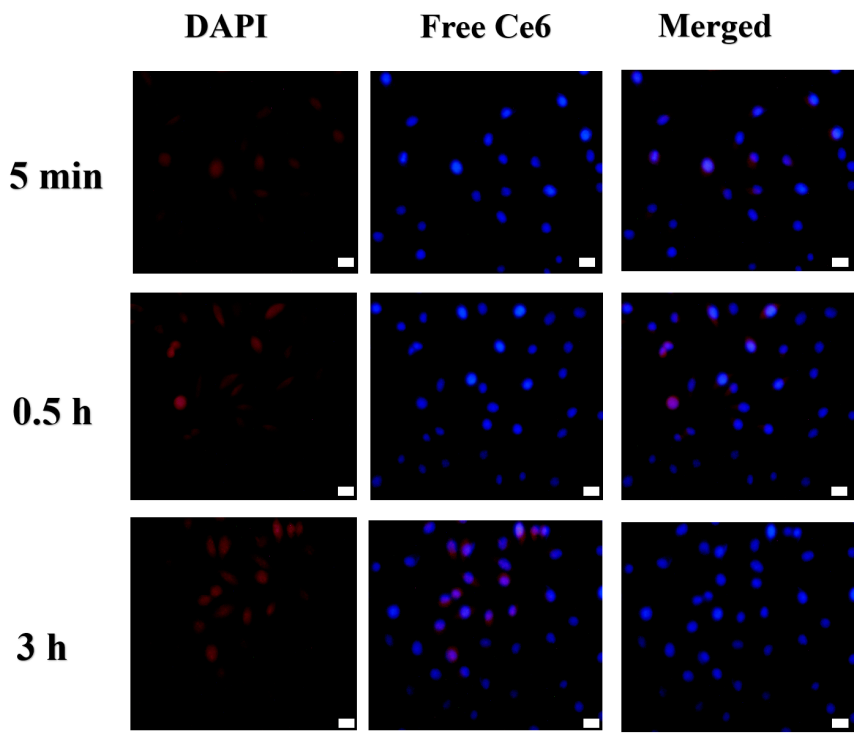


**Figure S15.** Free Ce6 (equivalent Ce6 concentration of 2 μg/mL) and L929 cells were incubated for 5 min, 30 min, and 3 h, respectively. A fluorescence inverted microscope was used for imaging, scale bar: 15 μm (blue for DAPI and red for NPs).


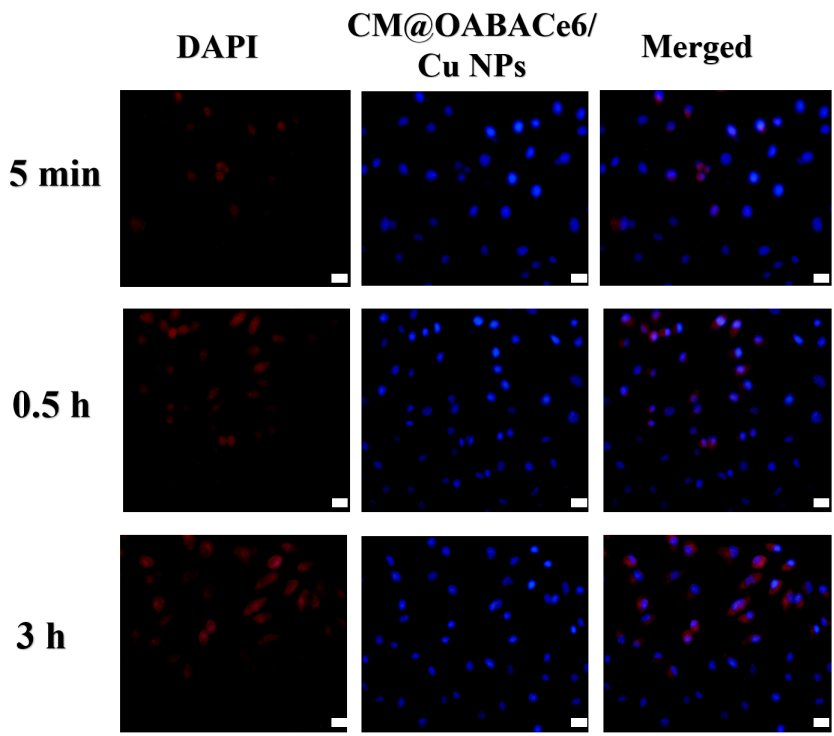


**Figure S16.** CM@OABACe6/Cu NPs (equivalent Ce6 concentration of 2 μg/mL) and L929 cells were incubated for 5 min, 30 min, and 3 h, respectively. A fluorescence inverted microscope was used for imaging, scale bar: 15 μm (blue for DAPI and red for NPs).


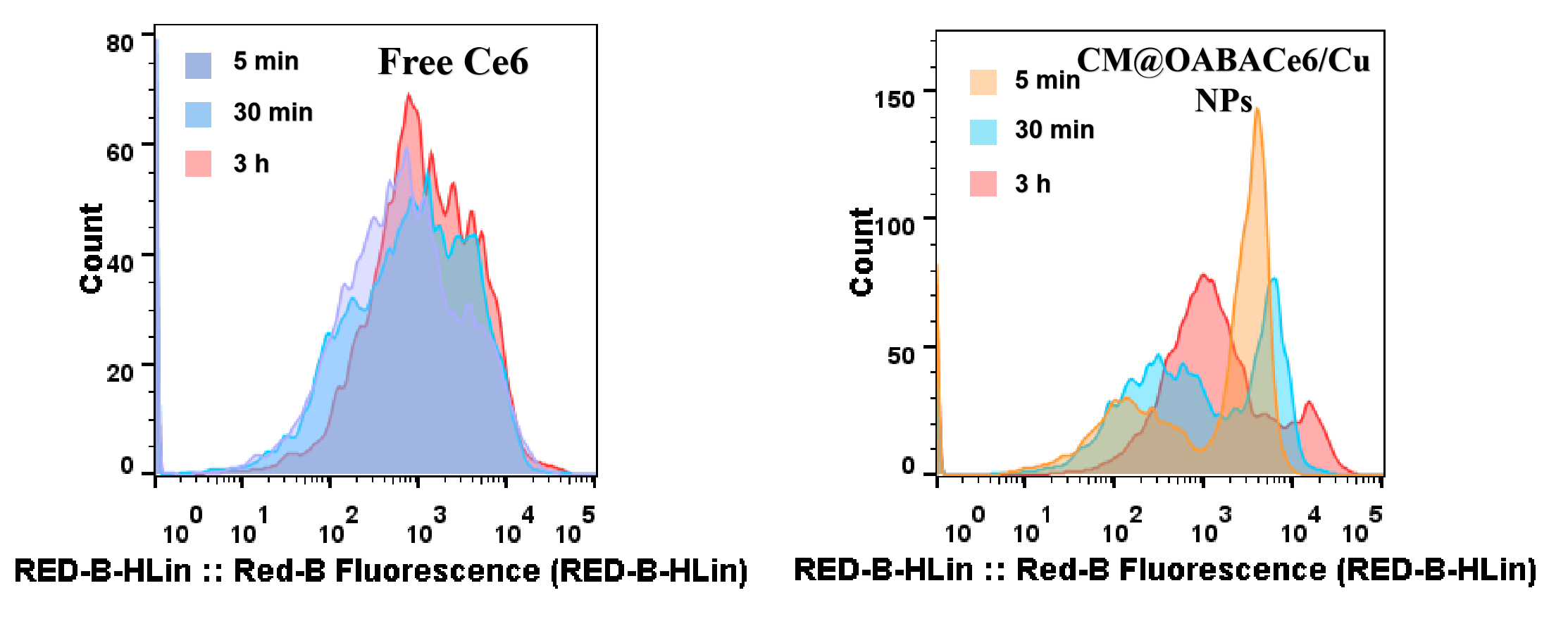


**Figure S17.** Free Ce6 and CM@OABACe6/Cu NPs (equivalent Ce6 concentration of 2 μg/mL) and L929 cells were incubated for 5 min, 30 min, and 3 h, respectively. The quantitative analysis was performed by flow cytometry.

**Table S6.** The mean fluorescence intensity of NPs.

| **Time** | **Nanoparticles** | |  |
| --- | --- | --- | --- |
|  | Free Ce6 | CM@OABACe6/Cu NPs | |
| 5min | 1713 | 2210 | |
| 0.5h | 1835 | 2465 | |
| 3h | 2253 | 3378 | |

**Table S7.** The IC30, IC50, IC70 analysis of free Ce6 and CM@OABACe6/Cu NPs against 4T1, respectively.

| Cell | Formulation | Ce6  (light) | CM@OABACe6/Cu NPs  (light) |
| --- | --- | --- | --- |
| 4T1 | IC30 | 3.663 | 1.014 |
|  | IC50 | 1.206 | 0.063 |
|  | IC70 | 0.397 | 0.004 |


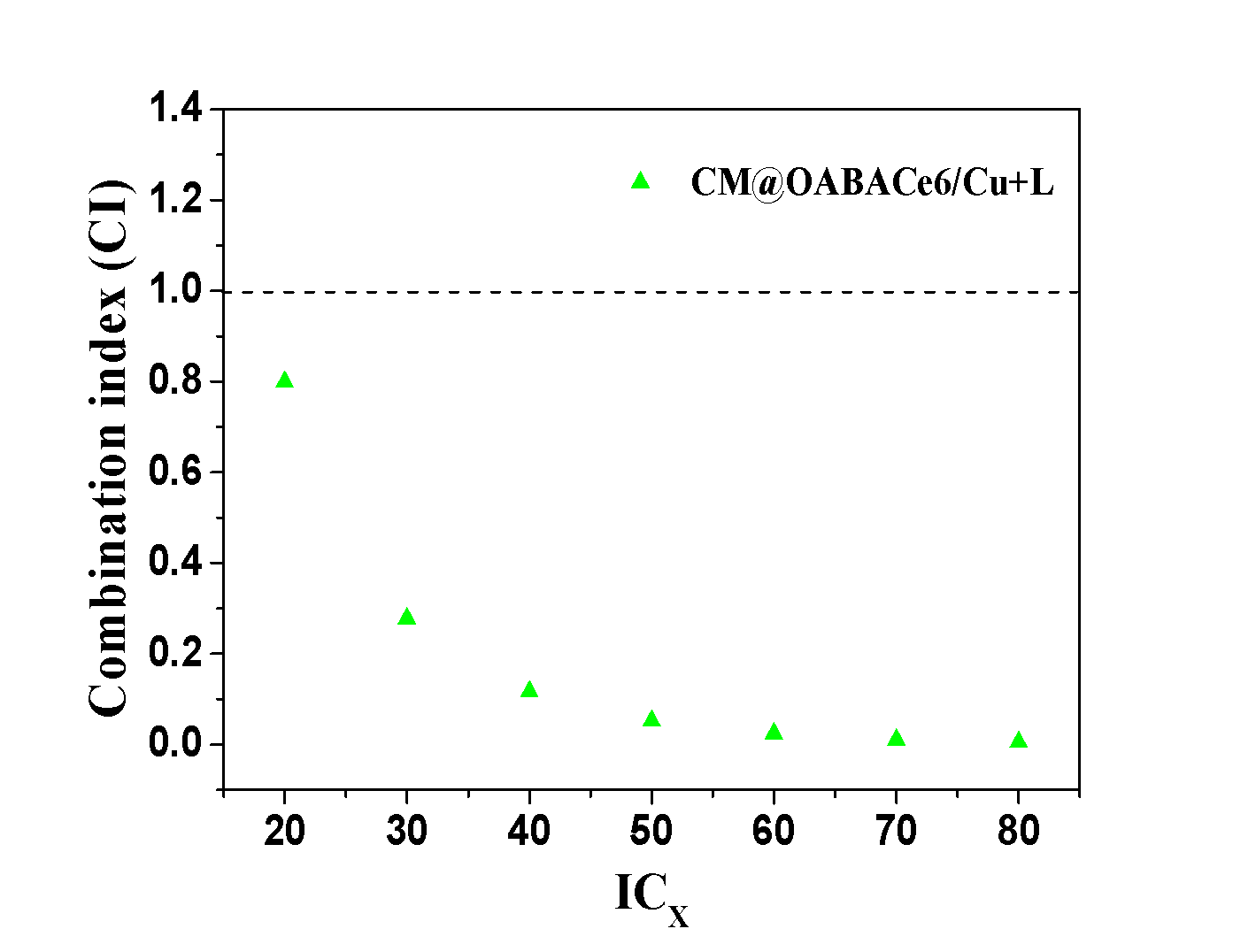


**Figure S18.** Combination index(CI) of CM@OABACe6/Cu NPs on 4T1 cells, as determined by ChouTalalay theorem calculation.


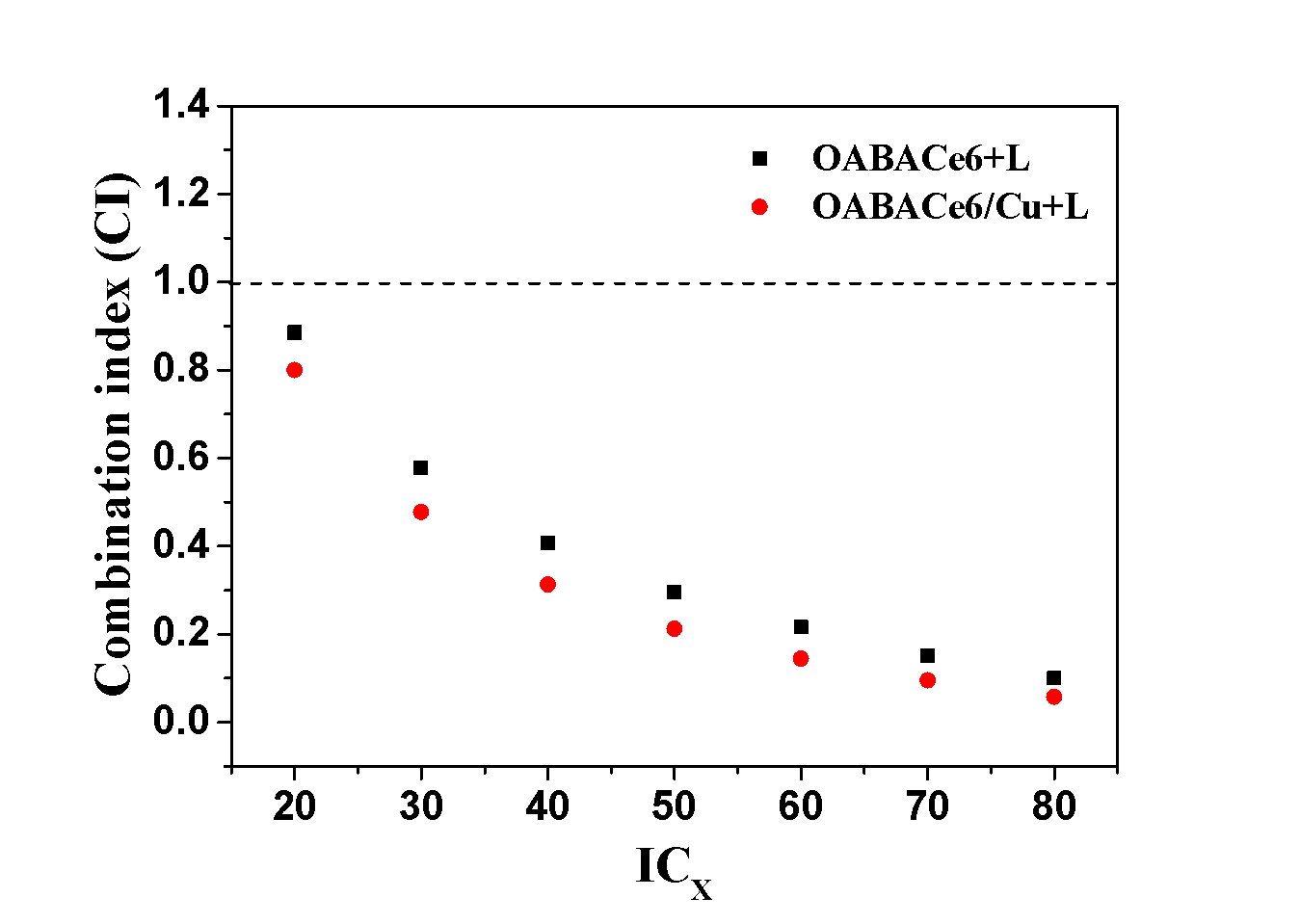


**Figure S19.** Combination index(CI) of OABACe6/Cu NPs and OABACe6 NPs on 4T1 cells, as determined by ChouTalalay theorem calculation.


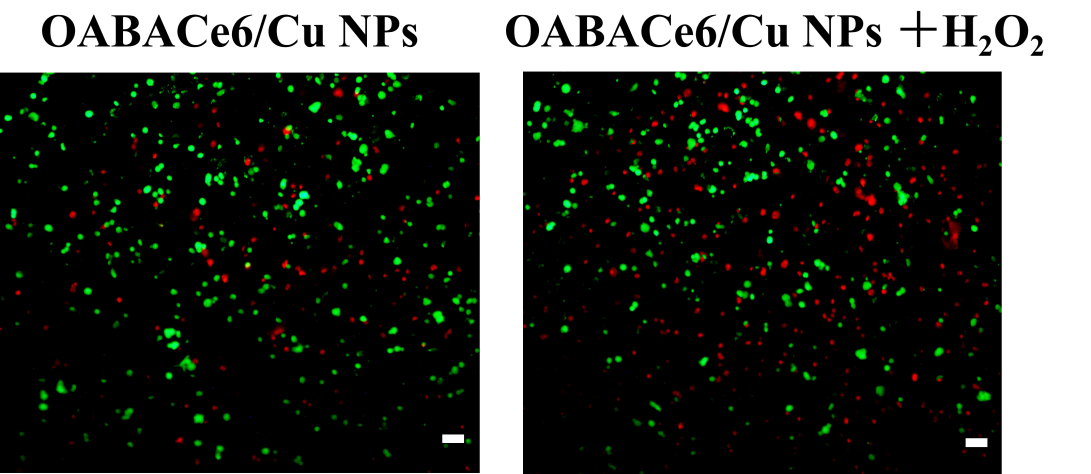


**Figure S20.** Calcein-AM/PI staining images of 4T1 cells after incubation with OABACe6/Cu NPs, and the mixture of OABACe6/Cu NPs, and H_2_O_2_, respectively, scale bar: 10 μm. (green for Calcein-AM and red for PI).


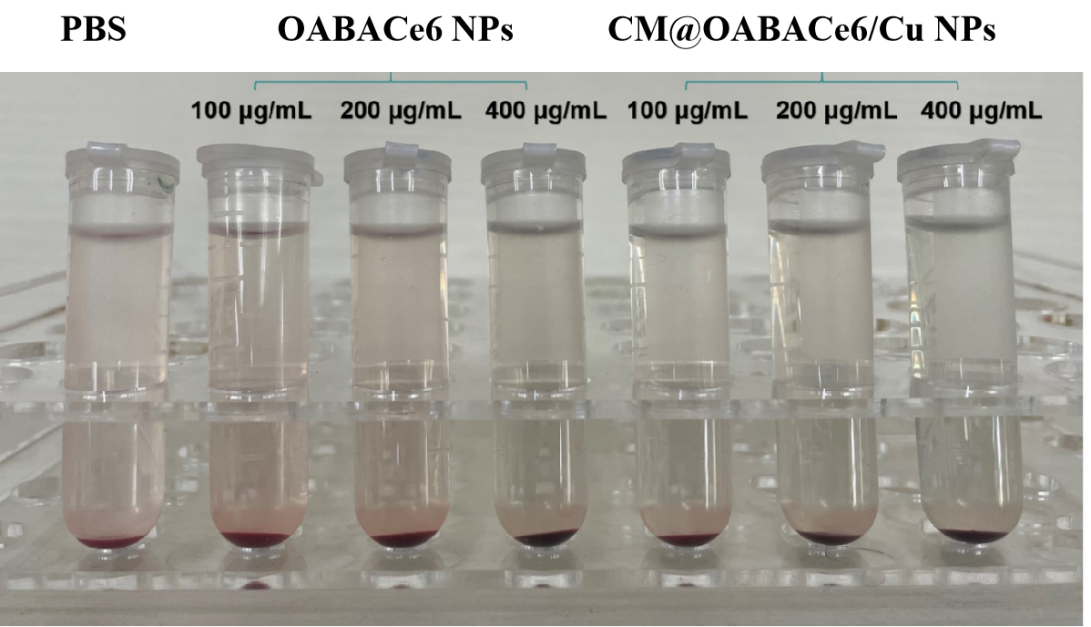


**Figure S21.** Photograph of hemolytic assay result of controls, OABACe6 NPs, and CM@OABACe6/Cu NPs at different concentrations (100, 200, and 400 μg/mL).


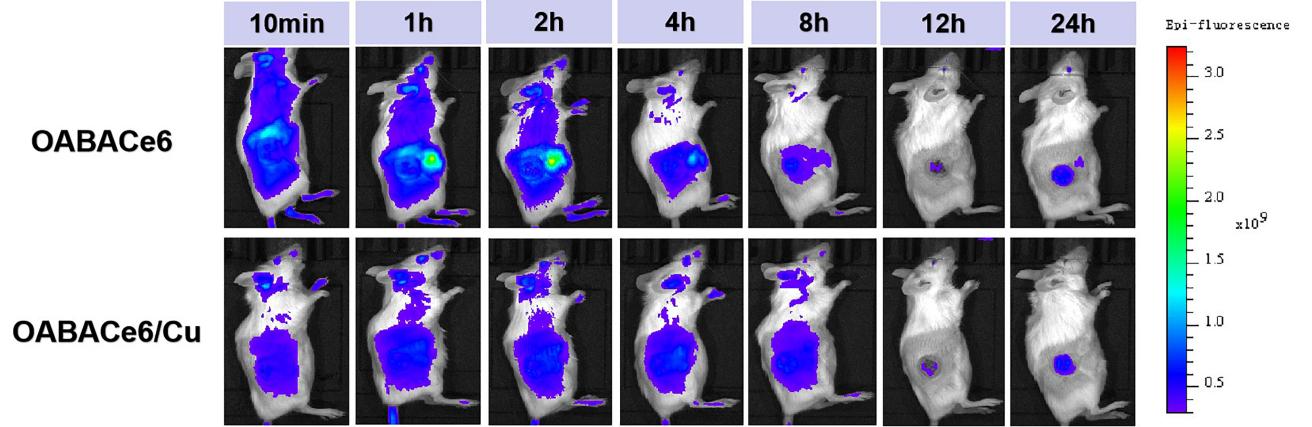


**Figure S22.** Imaging was performed at different time points after i.v. injection of OABACe6 NPs and OABACe6/Cu NPs. In vivo fluorescence imaging of 4T1 tumor-bearing.

**
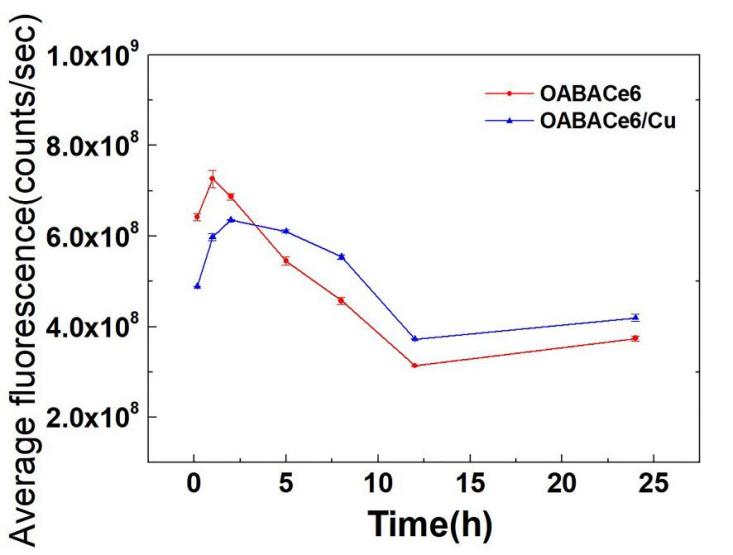
**

**Figure S23.** Imaging was performed at different time points after i.v. injection of OABACe6 NPs and OABACe6/Cu NPs. Average fluorescence intensity of 4T1 tumor-bearing mice.


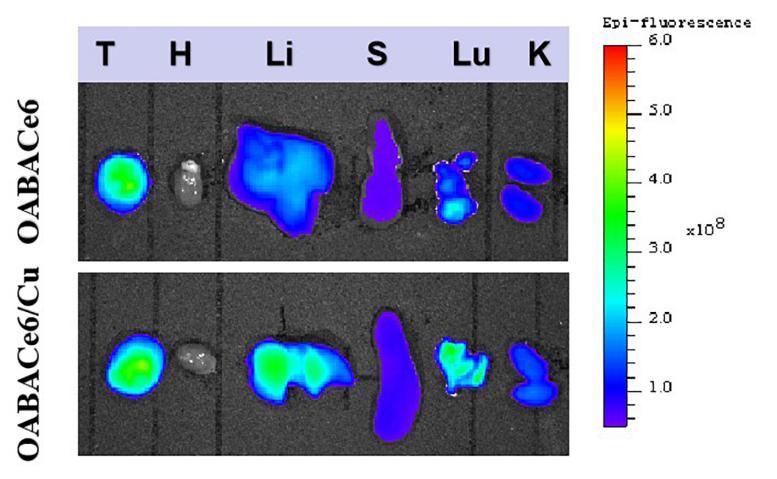


**Figure S24.** Imaging was performed at different time points after i.v. injection of OABACe6 NPs and OABACe6/Cu NPs. FL images of tumors and major organs (H: heart, Lu: lung, Li: liver, S: spleen, and K: kidneys) excised from mice after i.v.


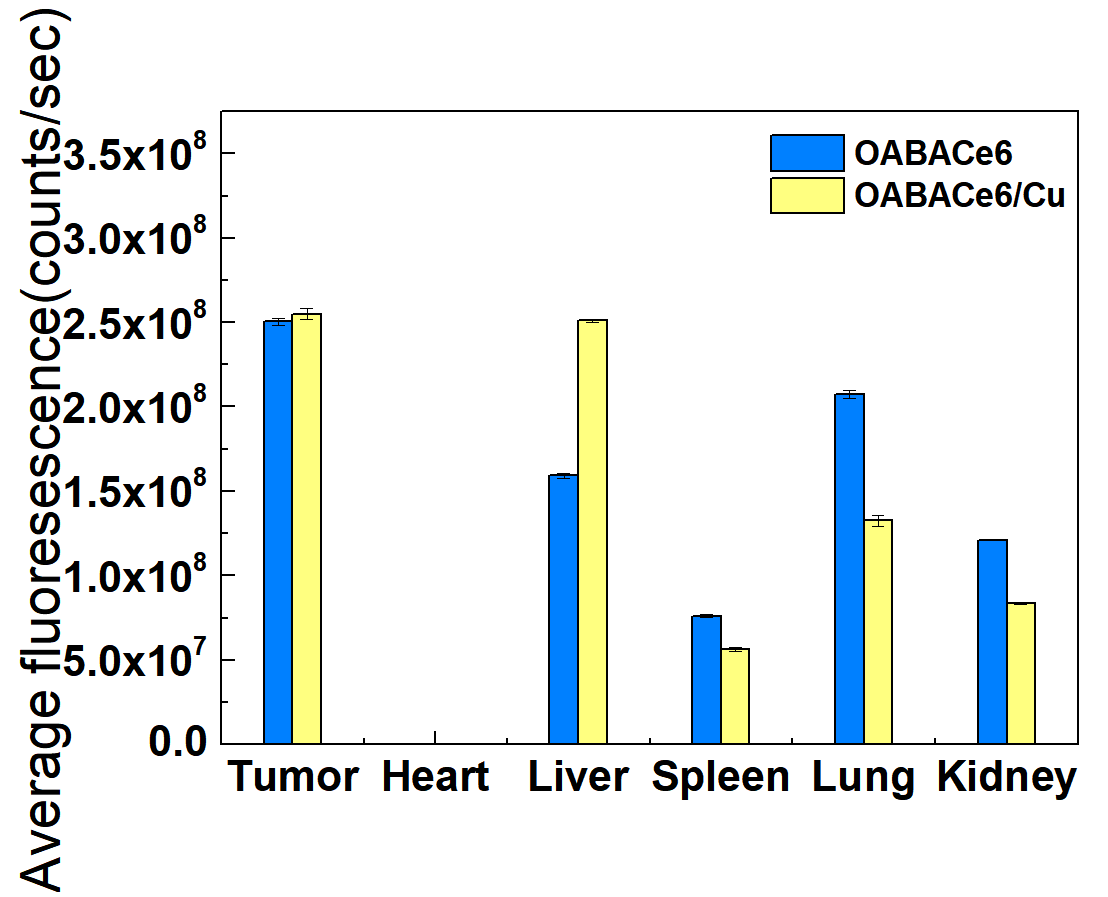


**Figure S25.** Imaging was performed at different time points after i.v. injection of OABACe6 NPs and OABACe6/Cu NPs. Mean fluorescence intensity of tumors and major organs (H: heart, Lu: lung, Li: liver, S: spleen, and K: kidneys) excised from mice after i.v.

**Figure S26.** *In vivo,* systematic studies focus on the optimization of dosing. The tumor inhibition rate after 12 days of PDT treatment at different doses (Ce6 equivalent: 2.5 mg/kg, 3.5 mg/kg, 4.5 mg/kg).


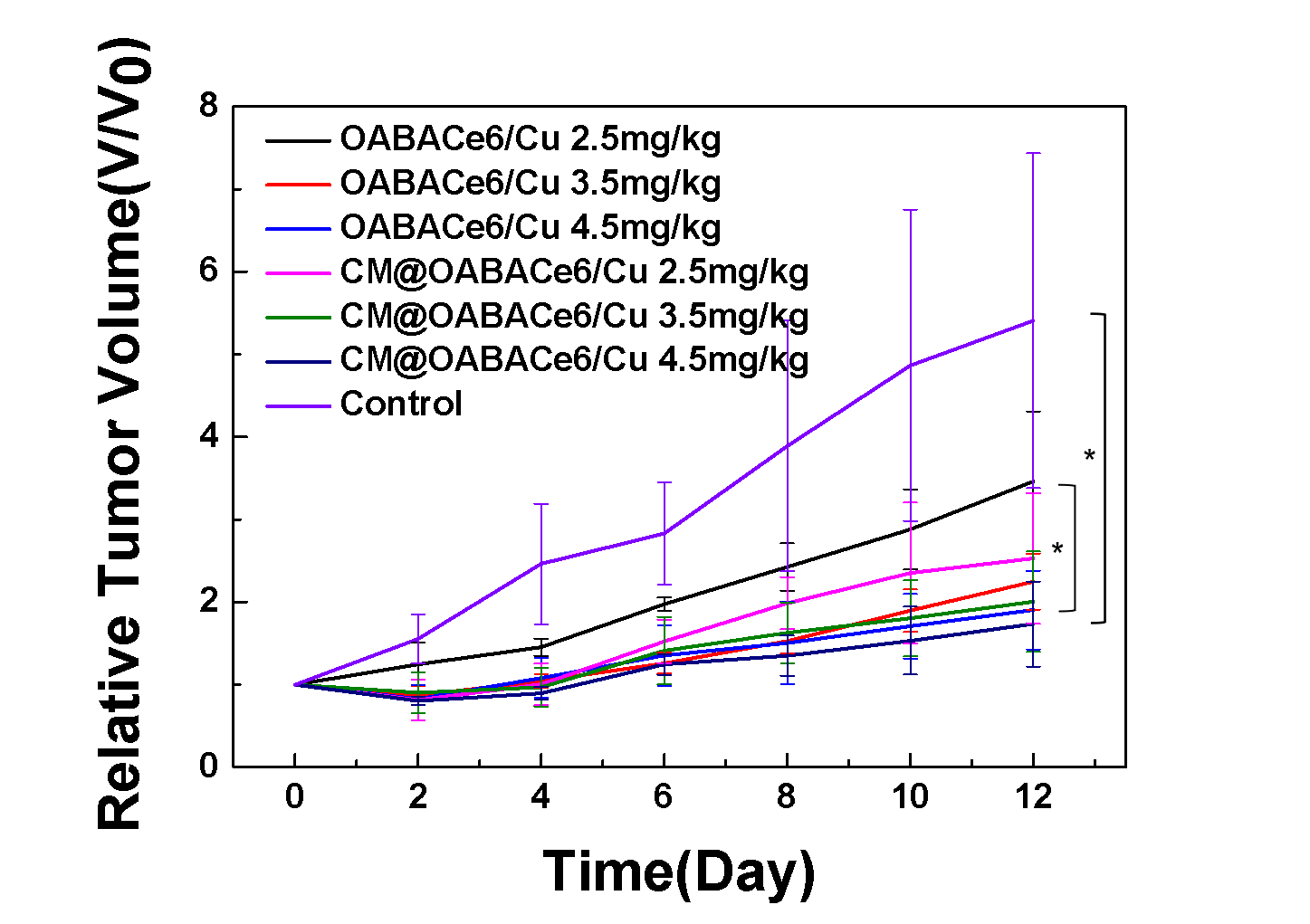


**Figure S27.** *In vivo*, systematic studies focus on the optimization of dosing. Relative tumor volume after 12 days of PDT treatment at different doses (Ce6 equivalent: 2.5 mg/kg, 3.5 mg/kg, 4.5 mg/kg). (*p<0.05 and **p<0.01, Student’s t test).


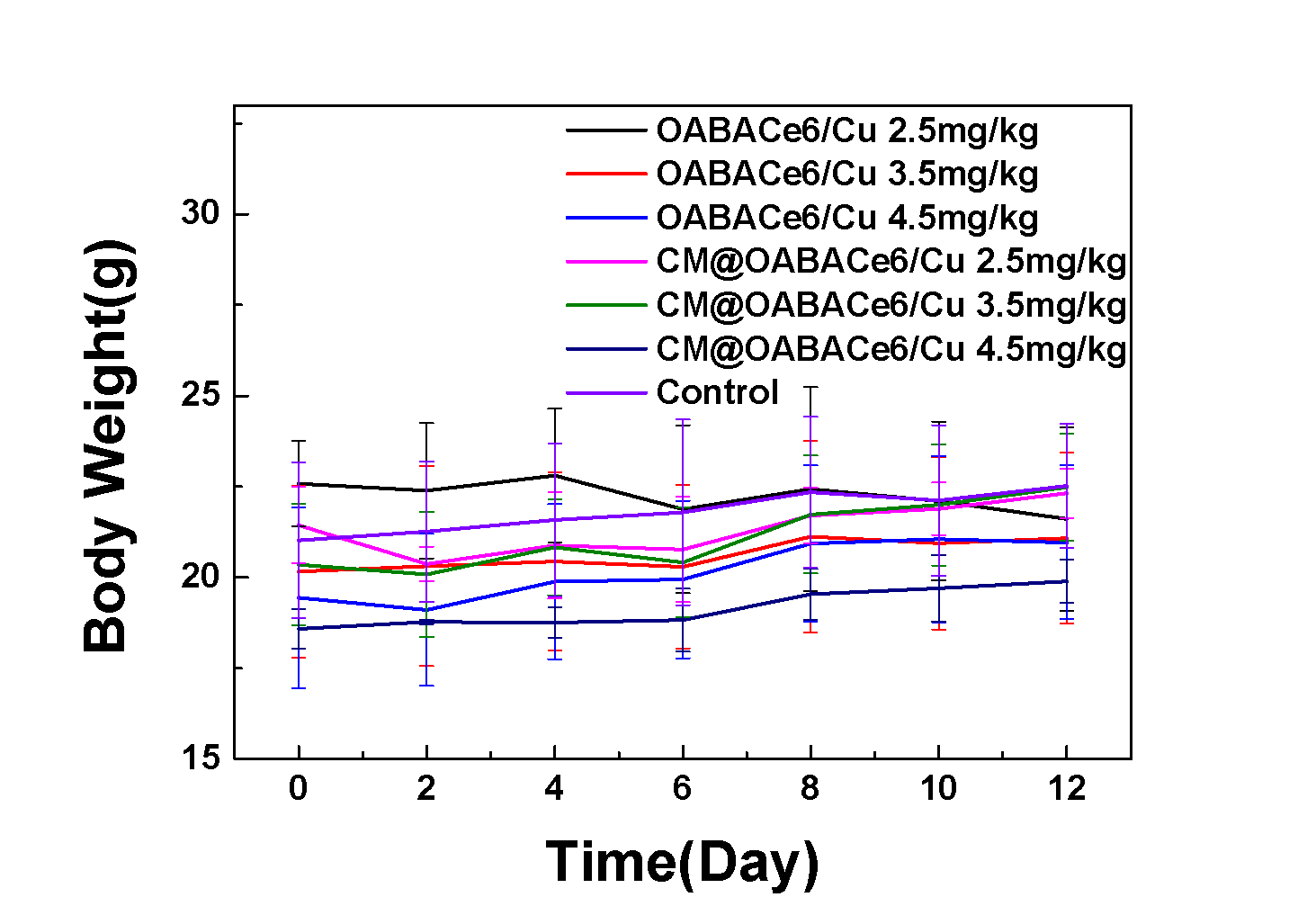


**Figure S28.** *In vivo*, systematic studies focus on the optimization of dosing. Weight changes were observed during 12 days of PDT treatment with different doses (Ce6 equivalent: 2.5 mg/kg, 3.5 mg/kg, 4.5 mg/kg).

**Figure S29.**Tumor weight in the eight treatment groups at the end of treatment.

**Figure S30.**The weight of each organ in the eight treatment groups after treatment.

**Declarations**

**Acknowledgments**

We greatly acknowledge the financial support from the National Natural Science Foundation of China (Project No. 31972040).

**Funding**

Not applicable.

**Author contributions**

XY and SYF conceived and designed the project. SYF carried out the experiments and data processing, MGW and BL performed data analysis and discussion. JJC, HTZ, HZ, AJD and WHL participated part of the experiments. XL and XY provided experimental drugs and quality control. All of the authors have read and approved the final manuscript.

**E-mail address:** yangxin@hit.edu.cn (**Corresponding author：**Xin Yang) .

**Authors' information**

Authors and Affiliations

^1^*School of Medicine and Health, Harbin Institute of Technology, No. 92, West Dazhi Street, Nangang District, Harbin, 150001, China.*

Shiyao Fu, Haitian Zhao, Weihong Lu, Xin Yang.

^2^*School of Chemistry and Chemical Engineering, Harbin Institute of Technology, No.92, West Dazhi Street, Nangang District, Harbin, 150001, China.*

Shiyao Fu, Jianjun Cheng, Hua Zhang, Aijun Dong, Xin Yang.

^3^*Academician Workstation, Jiangxi University of Traditional Chinese Medicine, No. 1088 Meiling Street, Wanli District, Nanchang, 330004, China.*

Mingao Wang.

^4^*Department of Nephrology, the First Affiliated Hospital of Harbin Medical University, No. 23 Youzheng Street, Nangang District, Harbin, 150001, China.*

Bin Li.

^5^*Department of Ophthalmology, the Second Hospital, Jilin University, No. 4026 Yatai street, Nanguan District, Changchun, 130041, China.*

Xu Li.

^6^*Chongqing Research Institute, Harbin Institute of Technology, No. 188 Jihuayuan South Road, Yubei District, Chongqing, 401135, China.*

Haitian Zhao, Weihong Lu, Xin Yang.

**Corresponding authors**

Xin Yang  **E-mail address:** yangxin@hit.edu.cn

**Ethics approval and consent to participate**

Not applicable.

**Consent for publication**

All authors have consented to the submission of this manuscript for publication.

**Competing interests**

The authors declare that they have no competing interests.
